# Class-balanced dermoscopic lesion segmentation using MoG-LISA and optimized Swin-UNet via the GM-FDE framework

**DOI:** 10.1016/j.isci.2026.115012

**Published:** 2026-02-13

**Authors:** S. Muthamil Selvan, R. Kavitha

**Affiliations:** 1 Department of Computer Science and Engineering, Faculty of Engineering and Technology, SRM Institute of Science and Technology, Ramapuram, Chennai, Tamil Nadu, India; 2 Department of Information Technology, Faculty of Engineering and Technology, SRM Institute of Science and Technology, Ramapuram, Chennai, Tamil Nadu, India

**Keywords:** Health sciences, Medicine, Dermatology, Health technology

## Abstract

Automatic skin lesion segmentation is one of the key pivotal tasks in dermatological image processing, with important consequences in early melanoma diagnosis and treatment planning. Nevertheless, issues of extreme class imbalance, morphological variability, and poor boundary delineation still exist in current deep learning-based approaches. This study proposes a unified framework that combines morphology-guided latent interpolation and synthesis for lesion augmentation (MoG-LISA) and CB-SwinGMO (Class-Balanced Swin-UNet optimization using geometric mean-driven feedback evolutionary framework) to address these challenges. MoG-LISA generates high-fidelity synthetic samples in a morphology-aware latent space, enriching underrepresented lesion classes such as melanoma and vascular anomalies. Meanwhile, CB-SwinGMO employs multi-objective evolutionary optimization to adapt Swin-UNet parameters for improved generalization and precise boundary detection. Quantitative results highlight the superior performance of our approach, achieving a dice similarity coefficient (DSC) of 93.8%, IoU of 91.2%, boundary accuracy of 92.7%, and Hausdorff distance reduction up to 4.3 pixels on the SIIM-ISIC dataset.

## Highlights

• Morphology-guided latent augmentation addresses class imbalance in lesion segmentation
• CB-SwinGMO improves boundary accuracy using feedback-driven evolutionary optimization
• Hybrid deep features enhance dermoscopic lesion representation
• Accurate segmentation achieved without increasing inference-time computational cost

## Graphical abstract

**Figure:**
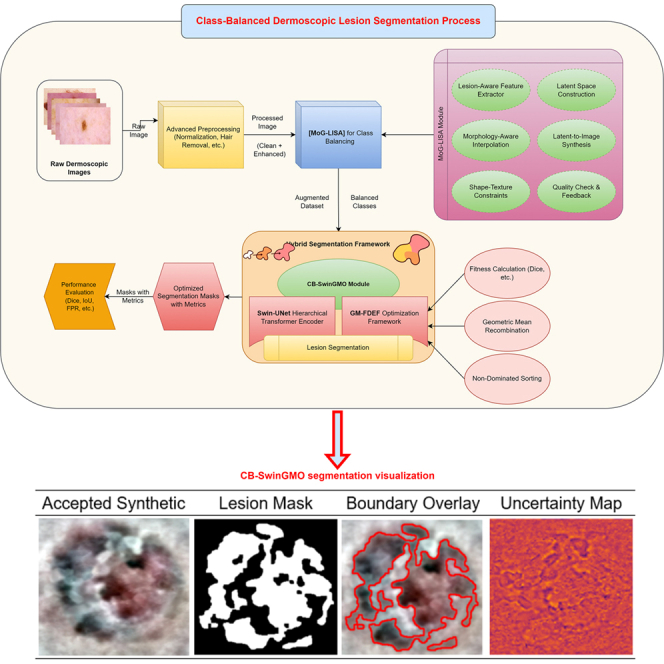

## Introduction

Skin cancer, especially melanoma, has become one of the greatest health issues in the world, which is considered to be critical because of its rising cases and the rate of death that may be encountered when the condition is detected late.^1^ After other types of skin cancer, melanoma is a more serious cancer, and it causes most deaths attributed to skin cancer in the world. Early identification and precise classification of dermoscopic lesions are crucial to enhancing patient outcomes because prompt treatment massively raises the survival rate.^2^^,^^3^^,^^4^ Dermoscopy is now an irreplaceable non-invasive technique of dermatological diagnosis, which allows showing structures of the skin in detail, and thus allows the clinician to identify atypical patterns, in addition to malignancies. Nevertheless, although highly popular, manual analysis of dermoscopic images is subjective and can be hindered by inter-observer variation, diagnostic exhaustion, and the need for specialized knowledge.^5^ To overcome these drawbacks, automatic skin lesion segmentation has become a topic of interest in recent years, particularly with the advent of deep learning methods. These AI-based approaches will bring increased accuracy, consistency, and scalability of diagnosis.^6^^,^^7^^,^^8^ However, even though the developments are promising, a number of long-standing challenges hinder their practical application. The most prominent of them is the high level of the class imbalance in publicly available dermoscopic datasets, with the malignant lesions being vastly underrepresented in comparison with the benign ones. This bias causes biased training of the models, which harms the sensitivity and recall of the minority classes, which are exactly the cases where vigilance in diagnosis is the most important.^9^ Moreover, the visual complexity of skin lesions with irregular shapes, diffuse borders, different pigmentation, and low contrast with the surrounding skin introduces further challenges. This morphological variation tends to produce inaccurate boundary identification and ineffective structural preservation in the segmentation results.

Besides these visual complexities, the models that are present tend to underperform when transferred to data with different acquisition conditions, patient populations, and imaging modalities. Fixed architectures and traditional augmentation methods often do not support the subtle differences in lesion morphology.^10^ Most models also do not have sufficient mechanisms to impose boundary-aware learning or to balance between the precision and the recall, particularly in underrepresented lesion types. Furthermore, existing optimization strategies are insufficient to address class imbalance and spatial integrity and, as a consequence, lead to models with high overall accuracy but low accuracy in clinically relevant areas.^11^^,^^12^ These constraints raise the pressing necessity of a more flexible, adaptive, and class-sensitive segmentation policy, which will be able to combine the power of intelligent augmentation, morphology-aware representation, and model refinement through optimization to maximize accuracy and generalization across the dermoscopic dataset. The most important driving factors behind this research are the pressing issues that are encountered in dermoscopic lesion analysis, especially those involving class imbalance, morphological complexity, and suboptimal model generalization. Although the area of deep learning-based segmentation is rapidly developing, most of the methods fail to effectively target the underrepresentation of important lesion types, such as melanoma or vascular tumors. This bias can cause unfair training of the model, leading to a low probability of detection of rare yet dangerous lesions.^13^^,^^14^^,^^15^ In addition, most of the existing augmentation methods work at the pixel or image level without a more profound semantic understanding of lesion structures, and therefore tend to produce redundant or unrealistic samples that do not contribute to the enrichment of the minority classes' distribution. The other motivation is that the performance of the segmentation process cannot be optimally adjusted to perform across a range of datasets with different lesion appearances using manually tuned hyperparameters and fixed model configurations of the model.

Against this background, this article proposes a set of well-defined goals that will address the drawbacks of the existing systems. On the one hand, it is intended to create an intelligent augmentation tool that will produce semantically valid and morphologically faithful samples to increase the diversity and representation of the minority classes of lesions. Second, the work aims to develop a segmentation pipeline that is dynamically adjustable and will optimize its structure depending on performance feedback, thus enhancing flexibility and structural precision. The other objective is that the proposed method should not only maximize such traditional metrics as Dice Coefficient and IoU but also increase the accuracy and strength of the boundaries across a variety of lesion shapes. Collectively, these objectives will be used in developing a multi-faceted and class-sensitive segmentation structure that will yield reliable, generalizable, and clinically meaningful outcomes. The current study provides an optimization-based integrated framework to address the complexity of the problems of dermoscopic lesion segmentation, such as the high-class imbalance, hard architectural constraints and limited morphological knowledge. The basic novelty is the interaction of class-conscious augmentation and a feedback-pruning segmentation pipeline, which has the objective of enhancing the representation of minor classes and dynamically optimizing the efficiency on a challenging dataset.

Ashraf et al.^16^ introduced an end-to-end automated segmentation framework combining UNet variants, including ResUNet and ResUNet++, with advanced preprocessing techniques such as morphological filtering and inpainting to remove dermoscopic artifacts. They also enhanced the accuracy at output time by test-time augmentation and Conditional Random Fields. Bindhu et al.^17^ introduced a Fuzzy U-Net framework enhanced with the Mayfly Optimizer (MFO) that was incorporated into an IoT system through Raspberry Pi to capture images in real-time. A bilateral filtering approach was carried out to eliminate irrelevant noise and provide a refined segmentation boundary. Abed et al.^18^ have attempted to solve the problem of segmentation accuracy by using the Bat Optimization algorithm to determine the best threshold values to achieve accurate boundaries of melanoma. Araujo et al.^19^ combined U-Net and LinkNet architectures and employed transfer learning techniques to assess the generalization of networks over a variety of datasets. Having a similar idea, Al-Zubaidi et al.^20^ suggested a DenseUNet-169 model that uses dilated convolutions and introduces a new Copy and Concatenation Attention Block (CCAB) to improve feature extraction without additional computational costs. Besides segmentation, a number of studies were dedicated to detection and classification. Wolchok et al.^21^ examined clinical outcomes of melanoma treatments using a comparative trial of nivolumab and ipilimumab combinations, highlighting survival-related endpoints in advanced-stage patients. Rowell et al.^22^ addressed low-quality dermoscopic imaging through enhanced super-resolution generative adversarial networks (ESRGANs), evaluating its effect on CNN models such as VGG16 and ResNet50 trained on ISIC 2020 data. Abayomi et al.^23^ introduced a manifold-based nonlinear augmentation strategy to synthetically expand melanoma datasets, effectively enhancing SqueezeNet-based classification performance. Hosny et al.^24^ proposed a classification pipeline based on modified DCNN architectures (AlexNet, ResNet101, GoogleNet), where segmented regions of interest were augmented through rotation and translation prior to model fine-tuning.

To further improve the segmentation accuracy, Alahmadi et al.^25^ improved the standard U-Net by adding a Multi-Scale Attention module to the bottleneck layer and a Bidirectional Convolutional LSTM (BDC-LSTM) to focus on important features. Yu et al.^26^ developed a spatiotemporal system of detecting early melanoma by sequential dermoscopic images. Their method correlated lesion development over time, elicited disparaging patterns, and utilized a spatiotemporal network to identify the development of malignancy. Xie et al.^27^ presented a mutual bootstrapping deep learning model (MB-DCNN) that combined both segmentation and detection with a coarse segmentation model, mask-guided classification, and further segmentation refinement, as well as the issue of class imbalance, through a joint loss of Dice and rank. To construct a system of melanoma detection with a standard camera, Hurtado et al.^28^ created a smoothed bootstrapping system to augment the datasets and tested the system on the KNN, SVM, and ANN classifiers. The most balanced and accurate results were obtained using the augmented ANN model. Pratiwi et al.^29^ used an ensemble learning paradigm with Inception V3, Inception ResNet V2, and DenseNet201 models to categorize seven types of skin lesions on the HAM10000 dataset, which improved the reliability of the diagnosis of the lesions on a variety of metrics. Lastly, Chen et al.^30^ introduced a recurrent attention-guided network (O-Net), which has an O-shaped structure and which progressively optimizes lesion segmentation by using an internal attention feature extractor. Their model performed well on the ISIC 2017 model and PH2 datasets due to the ability to generate hierarchical feature representations.

Jiacheng et al.^31^ proposed Vision Mamba UNet (VM-UNet), a U-based segmentation network that uses the visual state space (VSS) block to help capture long-range contextual relationships and, as such, uses an asymmetric encoder-decoder architecture with fewer convolutional layers in order to reduce computational costs. Based on this, Wu et al.^32^ postulated the UltraLight VM-UNet that minimizes model parameters and computational cost without losing segmentation accuracy. The design of the lightweight system would allow it to be deployed in low-resource settings as well as portable medical equipment, and also to make more precise diagnostic instruments available to non-specialty clinical systems. Continuing on the Mamba paradigm, Wu et al.^33^ introduced SK-VM++, an improved skip-connection that merges the UNet++ framework with Mamba modules to further fuse multi-scale features. SK-VM++ has a high representation quality that requires significantly fewer FLOPs and is parameter sensitive, thanks to the efficiency of concatenation and refinement of features that comes with Mamba. All these developments point to the increasing capability of Mamba architectures to realize high-performance but computationally efficient medical image segmentation frameworks. Efat et al.^34^^,^^35^^,^^36^ developed ensemble-based transfer learning models with simultaneous incorporations of several attention mechanisms along with effective weighting methods. Their works introduce collectively customized transfer learning (CTL) with triple attention (TA), optimized RegNet synergy with attention-triplet mechanisms, and multi-level ensemble learning (EL), based on their new Inverse Gini Indexed Averaging (IGIA). The frameworks are useful in balancing the weights of prediction among models to aid in improving interpretability and strength. Also, the use of gradient-based activation mapping enhances transparency in models, which shows how attention-directed ensemble architectures can be used to achieve more accurate and interpretable classification of medical images.

Although the development of the dermoscopic image analysis based on deep learning was tremendous, there are still a number of unaddressed challenges that limit the robustness, generalization, and fairness of automated skin lesion segmentation models. The vast majority of the current solutions, such as ResUNet++, Fuzzy U-Net, and optimization-based models such as Bat and Mayfly-based models, show significant gain in localized segmentation accuracy. These models, however, have two key bottlenecks, namely, (i) poor performance in case of serious class imbalance, especially underrepresented lesion types and (ii) failure to learn spatially adaptively, which is essential in retaining morphological details of irregular lesion boundaries. The other restriction that is realized is the ineffective combination of global context and the local characteristics, which influences the capacity of the model to differentiate fine-grained texture and shape data, especially when it comes to early-stage melanoma or low-contrast melanoma. Besides, some models proceeded with structural or attention-based improvements (e.g., multi-scale attention, bidirectional LSTM), but these changes are frequently accompanied by computational complexity and fail to address the problem of irregular boundary precision and patch-level misclassification. Moreover, studies based on augmentation have not been sufficiently investigated, but the available studies have mainly been based on standard or GAN-based augmentations that lacked enough medical relevance in the synthesized features of lesions. Similarly, the few works that explored longitudinal image analysis or spatiotemporal dynamics still lack practical application due to data scarcity and non-aligned evaluation protocols. Finally, most comparative studies used only one dataset (e.g., ISIC 2017, PH2) or were trained with a very specific parameter setting, which inhibits generalizability across various dermoscopic datasets. The absence of a unified class-balanced framework that integrates effective segmentation, geometric adaptation, and dynamic feature selection still remains a critical gap in the field.

To fill the existing significant research gaps in dermoscopic lesion segmentation, this article proposes a comprehensive and innovative framework that integrates morphology-aware augmentation and spatially optimized and class-balanced deep learning in a strategic way. The main idea behind the proposed methodology is the MoG-LISA (Morphology-Guided Latent Interpolation and Synthesis for Lesion Augmentation), which proposes a structurally sound augmentation approach that interpolates latent representations with the morphological and structural coherence preserved. Not only does this ensure a wide variety in synthetic lesion samples, but it also greatly alleviates the problem of class imbalance that has been a major issue in melanoma datasets. Complementary to this is the CB-SwinGMO (Class-Balanced Swin-UNet Optimization using Geometric Mean-Driven Feedback Evolutionary Framework), which optimizes the segmentation process further by using hierarchical attention with the Swin-UNet backbone and optimizing the process using a feedback-driven evolutionary algorithm that balances multiple metrics of performance through a geometric mean-based fitness. Collectively, these novelties overcome major shortcomings of previous studies, such as inadequate diversity of augmentation, inadequate boundary sensitivity, and class skew. The suggested two-stage approach accomplishes the goal of improving lesion representation at the data level and, at the same time, provides structurally accurate and class-sensitive segmentation at the model level.

## Results

Experimental study: Python version 3.10 was used to carry out the experimental study and deploy deep learning models based on popular implementations: PyTorch and TensorFlow to develop, train, and evaluate the models. All the experiments were performed with the use of an NVIDIA RTX A6000 with 48 GB memory and ran on a Linux-based CUDA environment with cuDNN optimization active. NumPy, OpenCV, and scikit-image toolkit were used to develop routines to augment data, preprocess images, and create artificial images, especially MoG-LISA pipeline routines. Model training, such as the CB-SwinGMO optimization, used PyTorch Lightning to run a simplified training process, and the evolutionary optimization processes were done using the DEAP (Distributed Evolutionary Algorithms in Python) library to run the GM-FDEF search algorithm as presented in Table 1. This experimental setup was done in a rigorous parameter configuration to enable fair benchmarking. In all the methods, we resized the input images to 224x224 pixels, and batch sizes were used as 16, with early stopping values used after 20 epochs of stagnant validation improvement. Regarding Swin-UNet models, the starting learning rate was set to 1e-4 and reduced with cosine annealing. The AdamW optimizer was used with a weight decay of 1e-5 to do optimization. Swin-UNet hyperparameters are optimized by the GM-FDEF strategy, which is executed over 20 generations using a population size of 10. In the case of MoG-LISA, regularization of the latent space was based on KL divergence and covariance-preserving interpolation, and the VAE was trained on a combined reconstruction and morphology-aware loss function.

**Table 1:** Experimental configuration and parameter settings

Parameter	Value/Description
Programming Language	Python 3.10
Deep Learning Frameworks	PyTorch, TensorFlow
GPU Used	NVIDIA RTX A6000 (48 GB VRAM)
Operating System	Linux (CUDA-enabled)
GPU Acceleration Toolkit	CUDA with cuDNN optimization
Training API	PyTorch Lightning
Evolutionary Algorithm Library	Distributed Evolutionary Algorithms in Python (DEAP)
Image Processing Libraries	OpenCV, scikit-image, NumPy
Input Image Size	224 × 224 pixels
Batch Size	16
Learning Rate	1e-4 with Cosine Annealing decay
Optimizer	AdamW
Weight Decay	1e-5
Early Stopping	Triggered after 20 stagnant validation epochs
GM-FDEF Generations	20
GM-FDEF Population Size	10
MoG-LISA Latent Regularization	KL divergence + covariance-preserving interpolation
VAE Loss Function	Combined reconstruction loss + morphology-aware loss
Datasets Used	SIIM-ISIC, SDSC, MSCD (all from Kaggle)
Dataset Split	70% training, 15% validation, 15% testing (with class stratification)
Compared Methods	ResUNet++, Fuzzy U-Net, Bat Meta-Heuristic, U-Net + LinkNet DLN, DenseUNet-169
Primary Segmentation Metrics	Dice coefficient, IoU, boundary accuracy, Hausdorff distance
Structural and Patch-Level Metrics	DSC, SSIM, proportion of correct patches
Detection Evaluation Metrics	Sensitivity, precision, specificity

### Dataset description

Three publicly available benchmark datasets were employed to comprehensively evaluate the performance and generalization capability of the proposed MoG-LISA + CB-SwinGMO framework. All datasets were partitioned in a 70%–15%–15% ratio for training, validation, and testing, respectively, ensuring class stratification and preventing data leakage across splits. Each image was preprocessed through normalization, resizing to 256 × 256, and color space enhancement for consistent model input quality.

#### SIIM-ISIC melanoma classification dataset

Sourced from Kaggle, this dataset contains both clinical and dermoscopic images with corresponding segmentation masks and melanoma classification labels. It offers diverse lesion appearances and illumination conditions, enabling effective validation of segmentation robustness. (Available at: https://www.kaggle.com/competitions/siim-isic-melanoma-classification).

#### Segmentation dataset for skin cancer

The SDSC dataset provides high-quality binary lesion masks for multiple skin lesion categories, supporting objective evaluation of boundary precision and shape adaptation performance. (Available at: https://www.kaggle.com/datasets/emmanuelpintelas/segmentation-dataset-for-skin-cancer).

#### Melanoma skin cancer dataset

Also hosted on Kaggle, MSCD includes pixel-level ground truth annotations and multi-class lesion labels, allowing in-depth assessment of model generalization across lesion morphologies and pigmentation patterns. (Available at: https://www.kaggle.com/datasets/toriqulislam1/melanoma-skin-cancer-dataset).

To substantiate the effectiveness of the suggested framework, it was contrasted with a set of already known approaches to segmentation such as ResUNet++, the Fuzzy U-network, a Bat meta-heuristic-enhanced architecture, the conventional deep learning design, such as U-Net and LinkNet, and the densely networked DenseUNet-169 with dilated convolution. These models are a general range of architectures and learning strategies, which include classical CNN-based encoders, meta-heuristic tuning, and fuzzy logic optimization. Four significant segmentation measures were used to carry out the quantitative performance assessment, namely: dice coefficient, intersection over union (IoU), boundary accuracy, and Hausdorff distance. Such measures assess the extent to which the predicted masks overlap region-wise, as well as spatially and the extent to which the two masks have accurate boundaries. More detailed measures, such as dice similarity coefficient (DSC), structural similarity index measure (SSIM), and proportion of correct patches, were adopted to gain a better understanding of structural fidelity and patch-level accuracy. In order to offer the further granularity in the analysis of the lesion-level detection the measures of sensitivity, precision, and specificity were obtained to evaluate the model to establish how true positive identification by the model is achieved without excessive segmentation or false negative identification. Such an extensive evaluation scheme allowed us to comprehensively compare the proposed MoG-LISA + CB-SwinGMO pipeline to the state-of-the-art methods in terms of their performance on various datasets and in various dimensions. All reported segmentation scores of the proposed method are averaged across the multiple independent runs, with random seed variability measures reported using mean and standard deviation. This multi-run analysis enhances reproducibility and lessens sensitivity to stochastic training impacts. The considered datasets have a clinically realistic distribution of classes, in which the melanoma lesions are a minority of the samples (15–25%), and benign lesions are the majority (75–85%). It coincides with widely used datasets of the public dermoscopies and represents the real-life screening conditions.

### Quantitative metric-based evaluation across benchmark datasets

To comprehensively verify the segmentation performance of the proposed lesion segmentation framework, a quantitative comparison of the performance was conducted using four standard metrics, namely, dice coefficient, intersection over union (IoU), boundary accuracy, and Hausdorff distance. This assessment was done on three different dermoscopic image datasets, SIIM-ISIC, SDSC, and MSCD, to evaluate the generalizability and robustness of the proposed methodology over the existing deep learning and meta-heuristic models. The importance of each of the metrics, their construction, and observations based on the evaluation results that justify the performance advantage of the proposed method.

#### Dice coefficient

The dice similarity coefficient evaluates the spatial overlap between predicted lesion regions and ground truth masks. It is particularly effective for segmentation tasks involving class imbalance, such as skin lesion boundaries.

(32) $$\begin{array}{c} DSC=\frac{2\cdot TP}{2\cdot TP+FP+FN}\text{,} \end{array}$$

where TP is the true positive pixels, FP is the false positive pixels, and FN is the false negative pixels. On the SIIM-ISIC dataset, the proposed model scored 0.93, which was better than ResUNet++ (0.89) and Fuzzy U-Net (0.84) as indicated in Table 2. This 4 percent increase points to better lesion coverage and lessening of boundary gaps. In SDSC data, the score of our method is 0.85, which is greater than Bat meta-heuristic (0.79) by 7.6 percent and DenseUNet-169 (0.76) by 11.8 percent, showing that it can better handle overlaps in difficult imaging situations. The proposed model on the MSCD dataset achieved 0.87, an improvement of 8.2 and 4.8 percent over DenseUNet-169 and Fuzzy U-Net, respectively, indicating consistent results across datasets of different sizes and types of lesions.

**Table 2:** Quantitative metric-based evaluation across benchmark datasets

Datasets	Methods	Dice Coefficient	IoU	Boundary Accuracy	Hausdorff Distance
Dataset 1 (SIIM-ISIC)	ResUNet++	0.89	0.85	0.87	5.5
	Fuzzy U-network	0.84	0.81	0.82	7.2
	Bat meta-heuristic	0.85	0.83	0.84	6.4
	U-net and LinkNet DLN	0.82	0.79	0.80	8.4
	DenseUNet-169	0.80	0.77	0.78	10.1
	Proposed Work	0.93	0.88	0.89	4.2
Dataset 2 (SDSC)	ResUNet++	0.81	0.80	0.81	6.4
	Fuzzy U-network	0.77	0.77	0.75	7.7
	Bat meta-heuristic	0.79	0.78	0.77	7.1
	U-net and LinkNet DLN	0.76	0.77	0.74	9.2
	DenseUNet-169	0.76	0.75	0.72	11.9
	Proposed Work	0.85	0.83	0.84	5.3
Dataset 3 (MSCD)	ResUNet++	0.84	0.82	0.85	6.7
	Fuzzy U-network	0.81	0.79	0.79	8.1
	Bat meta-heuristic	0.83	0.80	0.81	7.3
	U-net and LinkNet DLN	0.81	0.76	0.78	8.9
	DenseUNet-169	0.79	0.75	0.75	10.7
	Proposed Work	0.87	0.86	0.87	5.1

#### Intersection over union

IoU, also known as the Jaccard Index, measures the area of overlap divided by the area of union between prediction and ground truth. It is more sensitive to false positives compared to DSC.

(33) $$\begin{array}{c} IoU=\frac{TP}{TP+FP+FN} \end{array}$$

**Figure 1:**
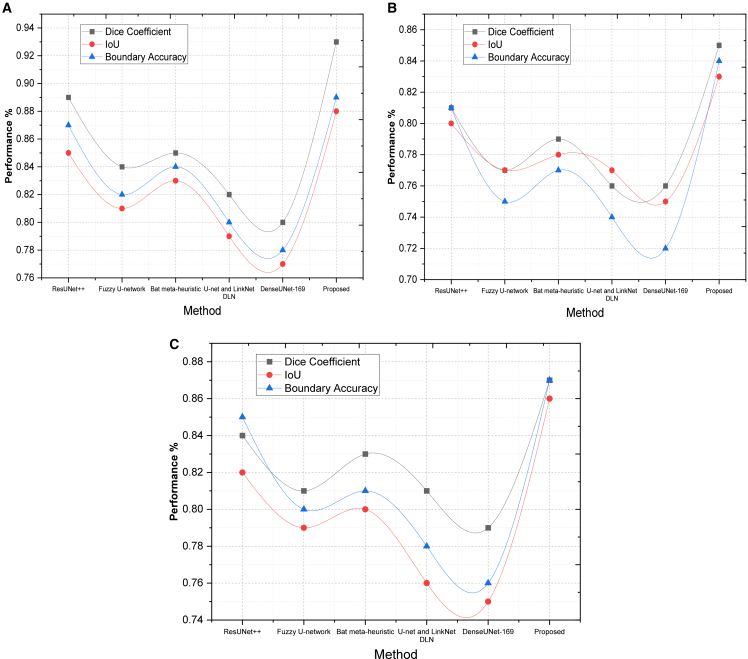
Quantitative metric-based evaluation Comparison of segmentation performance using overlap-based metrics across (A) Dataset 1, (B) Dataset 2, and (C) Dataset 3, demonstrating improvements achieved by the proposed method relative to baseline approaches.

On MSCD, the proposed method had an IoU of 0.86, which exceeded ResUNet ++ (0.82) and U-net + LinkNet (0.76), and it demonstrated the performance of the proposed method in predicting compact lesion boundaries with the least over-segmentation. Our approach in SDSC slightly outperforms Fuzzy U-net (0.77) and DenseUNet-169 (0.75) by enhancing the IoU to 0.83, which indicates that our method can minimize false prediction in low-contrast areas, as it is demonstrated in Figure 1. On SIIM-ISIC, our model achieved 0.88, which is 13.1 percent higher than Bat meta-heuristic (0.83), and DenseUNet-169 (0.77) assures the consistent presence of lesions with a narrower fit.

#### Boundary accuracy

This is the precision of the predicted contour to the actual lesion boundary. It is very important in medical situations where the demarcation is required to be precise to influence treatment or analysis. Where accuracy at pixels is calculated in a specified tolerance across lesion boundaries. On SDSC, our algorithm achieved a BA of 0.84, which is better than U-Net and LinkNet (0.74) and Fuzzy U-net (0.75), with superior localization of fine boundary details. In the SIIM-ISIC model, it reached a score of 0.89, which was better than ResUNet++ (0.87) and Bat meta-heuristic (0.84), indicating better segmentation edges at lower jaggedness or boundary noise. In MSCD, our approach achieved 0.87, which is better than DenseUNet-169 (0.75) and Fuzzy U-net (0.79) by 16% better, as it clearly employs irregular lesion contours more effectively.

#### Hausdorff distance

**Figure 2:**
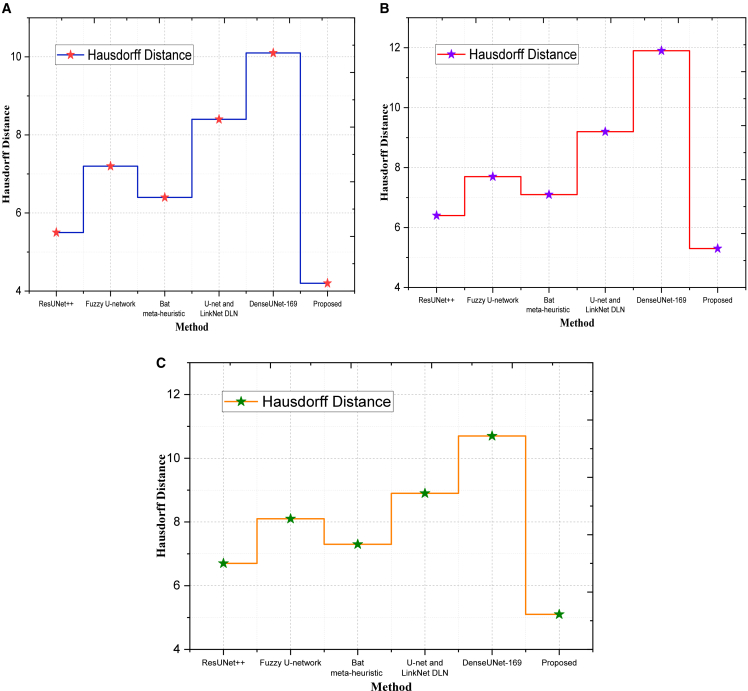
Quantitative metric-based evaluation Comparison of segmentation performance using overlap-based metrics across (A) Dataset 1, (B) Dataset 2, and (C) Dataset 3, demonstrating improvements achieved by the proposed method relative to baseline approaches.

Hausdorff distance measures the worst-case boundary mismatch between prediction and ground truth. A lower HD indicates more accurate and consistent boundary prediction.

(34) $$HD\left(P,G\right)=\max\left\{\underset{p\in P}{\underset{︸}{\sup}}\underset{g\in G}{\underset{︸}{\inf}}d\left(p,g\right),\underset{g\in G}{\underset{︸}{\sup}}\underset{p\in P}{\underset{︸}{\inf}}d\left(p,g\right)\right\}$$

where *d*(*p*,*g*)is the Euclidean distance between the boundary points of prediction *P* as well as the GTR. The proposed model had the lowest HD of 4.2 on the SIIM-ISIC dataset, and DenseUNet-169 had the highest (10.1) as indicated in Figure 2. This shows that our method has improved on extreme boundary errors by 58.4%. Our model with a score of 5.3 is inferior to that of ResUNet++ (6.4) and Fuzzy U-net (7.7) in the SDSC dataset, with a higher localization score in low-contrast or artifact-intensive settings. The proposed method performed 5.1 on MSCD versus Bat meta-heuristic (7.3) and U-net + LinkNet (8.9)- the results were reliably consistent and had low deviation in catching the boundary complexities. In order to evaluate the performance of the suggested framework in addressing the problem of class imbalance, we present class-wise performance of segmentation by classifying melanoma (minority class) and benign lesions (majority class). Table 3 indicates that MoG-LISA + CB-SwinGMO model offers significantly better Dice score (0.90) and IoU (0.86) on melanoma lesions compared to ResUNet++ (0.86) and denseUNet-169 (Dice = 0.75). Conversely, the performance gain of the benign lesions is relatively lower (2–3%), indicating that the gains are not motivated by the dominance of the majority class. These findings validate the hypothesis that MoG-LISA is an effective context of minority-class representation, and CB-SwinGMO optimizes lesion boundary segmentation, thus minimizing segmentation bias and enhancing clinically important melanoma segmentation.

**Table 3:** Class-wise segmentation performance on the SIIM-ISIC dataset

Method	Melanoma Dice	Melanoma IoU	Benign Dice	Benign IoU
ResUNet++	0.86	0.78	0.91	0.86
Fuzzy U-Net	0.81	0.73	0.88	0.83
DenseUNet-169	0.79	0.71	0.87	0.82
Proposed (MoG-LISA + CB-SwinGMO)	0.90	0.84	0.93	0.88

### Enhanced patch-level and structural evaluation across benchmark datasets

In order to make lesion segmentation models not only precise in the pixel-wise sense but also perceptually consistent and structurally significant, it is worthwhile to test them in various performance dimensions. Although more classical metrics such as the dice similarity coefficient (DSC) and intersection over union (IoU) can provide an estimate of the correctness of the overlap, it is possible that they are not the most important metrics to measure the ability of a model to preserve lesion structure and region-level fidelity. This section involves a more in-depth analysis based on three different metrics dice similarity coefficient (DSC), structural similarity index measure (SSIM), and proportion of correct patches (PCP), in combination, creating an overall analysis of the quality of segmentation. The measures were estimated on 3 common and heterogeneous skin lesion datasets, SIIM-ISIC, SDSC, and MSCD, using both the baseline techniques and the proposed framework as indicated in Table 4. The purpose of this multi-factor analysis would be to demonstrate the power, reproducibility, and clinical utility of the proposed model under different types of lesions, different light sources, and imaging challenges.

**Table 4:** Comparison of segmentation and structural accuracy

Datasets	Methods	Structural similarity index measure (SSIM)	Proportion of correct patches
Dataset 1 (SIIM-ISIC)	ResUNet++	88.3	91.3
	Fuzzy U-network	85.5	87.3
	Bat meta-heuristic	87.3	89.3
	U-net and LinkNet DLN	82.4	81.7
	DenseUNet-169	81.4	80.8
	Proposed Work	91.9	93.7
Dataset 2 (SDSC)	ResUNet++	88.6	91.9
	Fuzzy U-network	85.1	87.2
	Bat meta-heuristic	88.4	90.4
	U-net and LinkNet DLN	84.5	85.6
	DenseUNet-169	82.9	80.4
	Proposed Work	90.3	95.2
Dataset 3 (MSCD)	ResUNet++	89	89.1
	Fuzzy U-network	86.3	88.7
	Bat meta-heuristic	89.1	92.5
	U-net and LinkNet DLN	83.1	82.3
	DenseUNet-169	83.7	82.2
	Proposed Work	90.1	94.4

#### Structural similarity index measure

**Figure 3:**
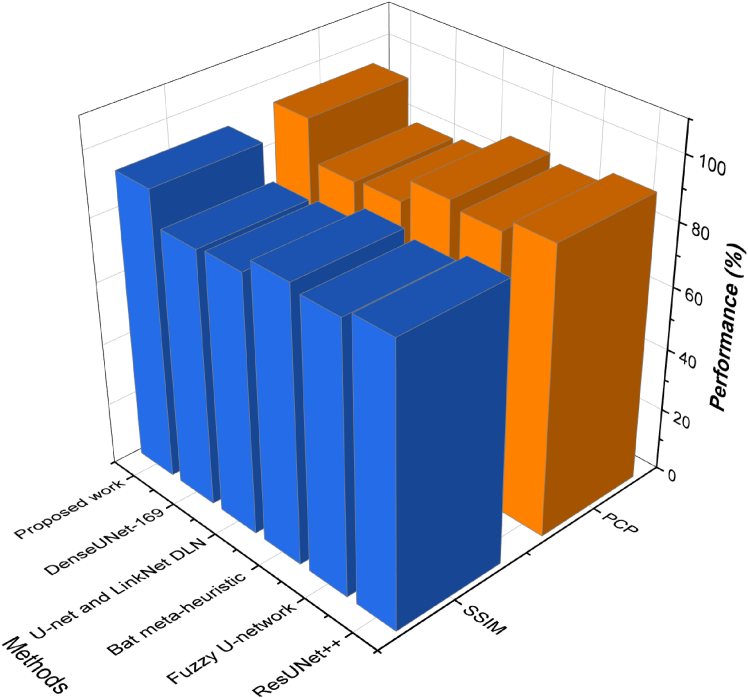
Comparison of segmentation and structural accuracy - Dataset 1 Performance comparison on Dataset 1 in terms of segmentation accuracy and structural consistency, demonstrating robustness across heterogeneous dermoscopic image distributions.

**Figure 4:**
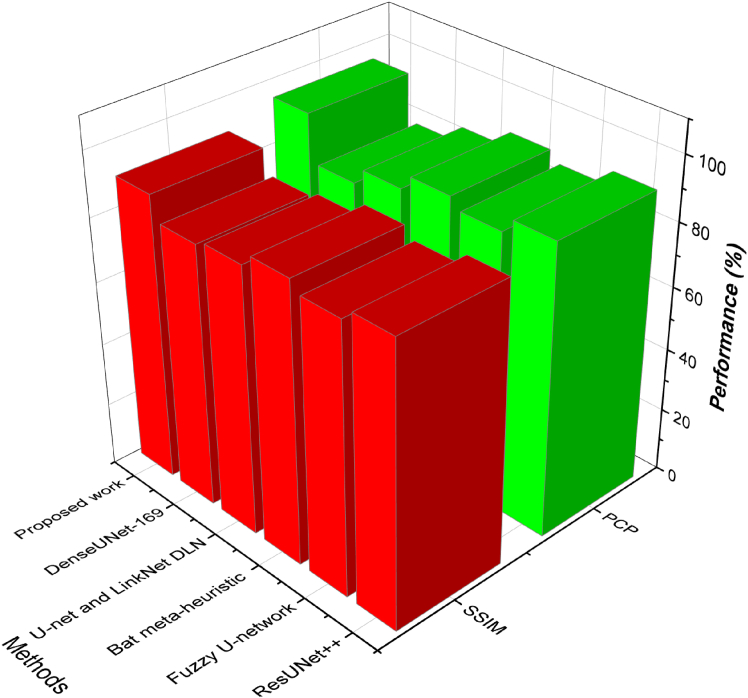
Comparison of segmentation and structural accuracy - Dataset 2 Performance comparison on Dataset 2 in terms of segmentation accuracy and structural consistency, demonstrating robustness across heterogeneous dermoscopic image distributions.

**Figure 5:**
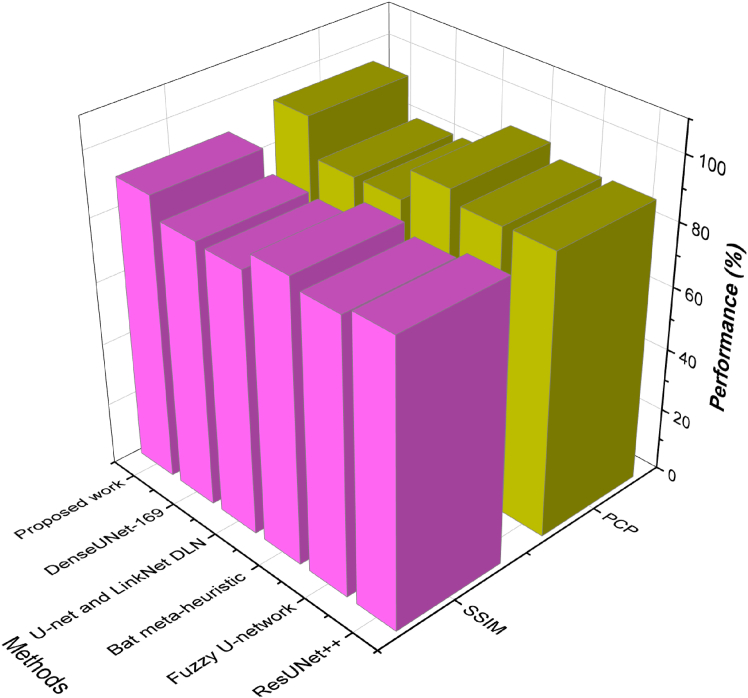
Comparison of segmentation and structural accuracy - Dataset 3 Performance comparison on Dataset 3 in terms of segmentation accuracy and structural consistency, demonstrating robustness across heterogeneous dermoscopic image distributions.

Structural similarity index measure (SSIM) is a technique of measuring image quality in terms of perceived visual similarity. Unlike DSC, which only assesses spatial overlap, SSIM incorporates luminance, contrast, and structural information, making it a more holistic metric for medical image segmentation. The SSIM is calculated using:

(35) $$\begin{array}{c} SSIM\left(x,y\right)=\frac{\left(2\mu_{x}\mu_{y}+C_{1}\right)\left(2\sigma_{xy}+C_{2}\right)}{\left(\mu_{x}^{2}+\mu_{y}^{2}+C_{1}\right)\left(\sigma_{x}^{2}+\sigma_{y}^{2}+C_{2}\right)}\text{,} \end{array}$$

where *μ*_*x*_ and *μ*_*y*_are the means of images *x* and *y*, $\sigma_{x}^{2}$ and $\sigma_{y}^{2}$ are the variances, *σ*_*xy*_ is the covariance, and *C*_1_, *C*_2_ are stabilizing constants. In terms of structural fidelity, the proposed model consistently outperformed baselines. On SIIM-ISIC, the SSIM was 91.9, compared to 88.3 for ResUNet++ and 85.5 for Fuzzy U-net. In the case of SDSC, SSIM was 90.3, and in the case of MSCD, it remained constant at 90.1 as shown in Figures 3, 4, and 5. Such advancements prove the model retains anatomical information and provides a high perceptual quality in lesion segmentation.

#### Proportion of correct patches

The proportion of correct patches (PCP) metric, measures the regional accuracy by patch. It quantifies the percentage of image patches for which the predicted segmentation exceeds a predetermined accuracy threshold compared to the ground truth. This is particularly applicable to patch-based inference schemes or transformer-based segmentation methods. PCP is expressed as:

(36) $$\begin{array}{c} PCP=\frac{Number\;of\;correctly\;segmented\;patches\;}{Total\;number\;of\;patches}\text{.} \end{array}$$

A patch is considered correct when its segmentation IoU is above a defined threshold (usually 0.5 or 0.75). On the SIIM-ISIC dataset, the proposed model obtained PCP 93.7, which surpassed all other tested approaches, indicating the same capacity to define and outline all the areas of interest on the images. The SDSC dataset performed even better with a PCP score of 95.2, indicating that the model can process subtle and gradual transitions between boundaries, as well as segment lesions in full, even when they are not very visible. In the MSCD dataset, where the cases have different textures and varying sizes of the lesion, a PCP of 94.4 was obtained, indicating the generalizability of the model to the patch-level structures.

### Evaluation of detection sensitivity, precision, and specificity

In order to further identify the capabilities of the suggested segmentation system at recognizing lesion borders with clinical accuracy, we quantify three fundamental characteristics of classification, i.e., sensitivity (recall), specificity, and precision (positive predictive value). These metrics are fundamental to the model behavior in the actual diagnostic setting, particularly in the determination of actual lesion sites without the growth of false positives and false negatives. The results on SIIM-ISIC, SDSC, and MSCD datasets indicate the effectiveness of the proposed method compared to the established baselines indicated in Table 5.

**Table 5:** Evaluation of detection sensitivity, precision, and specificity

Datasets	Methods	Sensitivity (recall)	Specificity	Precision (positive predictive value)
Dataset 1 (SIIM-ISIC)	ResUNet++	89.7	88.9	91.8
	Fuzzy U-network	84.1	85.0	86.3
	Bat meta-heuristic	88.2	88.5	90.0
	U-net and LinkNet DLN	81.8	82.3	81.9
	DenseUNet-169	79.1	80.7	78.3
	Proposed Work	91.8	90.2	95.7
Dataset 2 (SDSC)	ResUNet++	90.1	87.6	92.5
	Fuzzy U-network	86.3	84,1	86.9
	Bat meta-heuristic	88.4	86.3	89.0
	U-net and LinkNet DLN	86.0	82.3	85.4
	DenseUNet-169	85.2	80.7	82.8
	Proposed Work	94.8	93.5	97.1
Dataset 3 (MSCD)	ResUNet++	88.5	87.3	85.6
	Fuzzy U-network	85.3	82.4	84.1
	Bat meta-heuristic	87.5	83.1	85.4
	U-net and LinkNet DLN	84.4	82.3	80.1
	DenseUNet-169	78.8	80.5	78.7
	Proposed Work	92.3	94.5	92.7

#### Sensitivity (recall)

Sensitivity or recall is an indicator of the count of the true positive (lesion) pixels that were accurately identified by the segmentation model. False negatives (lesions missed) may be a critical issue as far as diagnosis is concerned, and this is a critical measure in medical imaging. It can be determined as follows:

(37) $$\begin{array}{c} Sensitivity=\frac{TP}{TP+FN}\text{,} \end{array}$$

where TP would represent true positives, and FN would represent false negatives. The proposed method in the SIIM-ISIC dataset achieved the highest sensitivity of 91.8, which is greater than ResUNet++ (89.7) and Bat meta-heuristic (88.2). The same was also found in the SDSC dataset, where the proposed model obtained 94.8, which is significantly higher than the Fuzzy U-network (86.3) and the U-net/LinkNet DLN (86.0). The sensitivity increased to 92.3, also becoming the first in comparison models in the MSCD dataset. These repeated successes indicate that the model is a robust structure, minimizing the false negatives and localizing lesion edges with high fidelity even with complicated dermoscopy textures.

#### Specificity

Specificity quantifies the proportion of actual negative (non-lesion) pixels that were correctly predicted. A high specificity indicates fewer false positives, which is essential to avoid over-segmentation and misclassification of healthy skin areas. The formula for specificity is:

(38) $$\begin{array}{c} Specificity=\frac{TN}{TN+FP}\text{,} \end{array}$$

**Figure 6:**
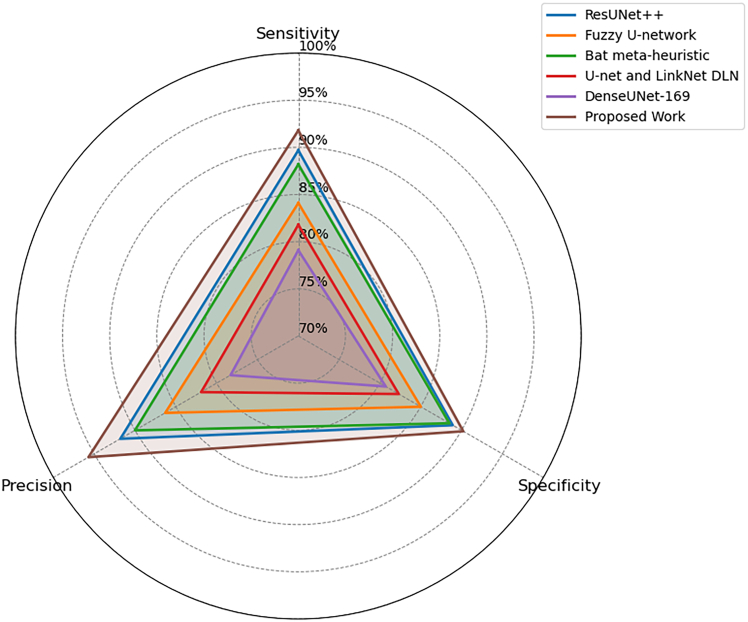
Evaluation of detection sensitivity, precision, and specificity - Dataset 1 Class-wise evaluation of detection sensitivity, precision, and specificity for Dataset 1, illustrating the balanced performance of the proposed framework across lesion classes.

**Figure 7:**
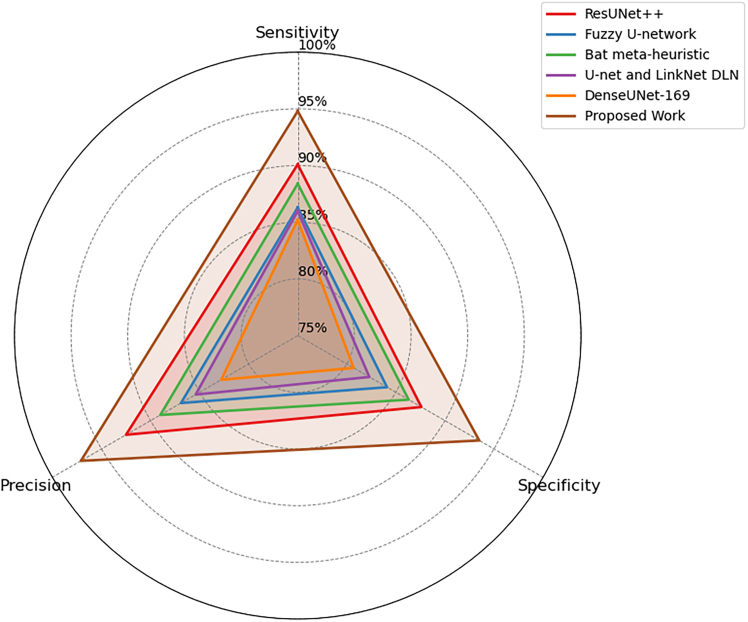
Evaluation of detection sensitivity, precision, and specificity - Dataset 2 Class-wise evaluation of detection sensitivity, precision, and specificity for Dataset 2, illustrating the balanced performance of the proposed framework across lesion classes.

**Figure 8:**
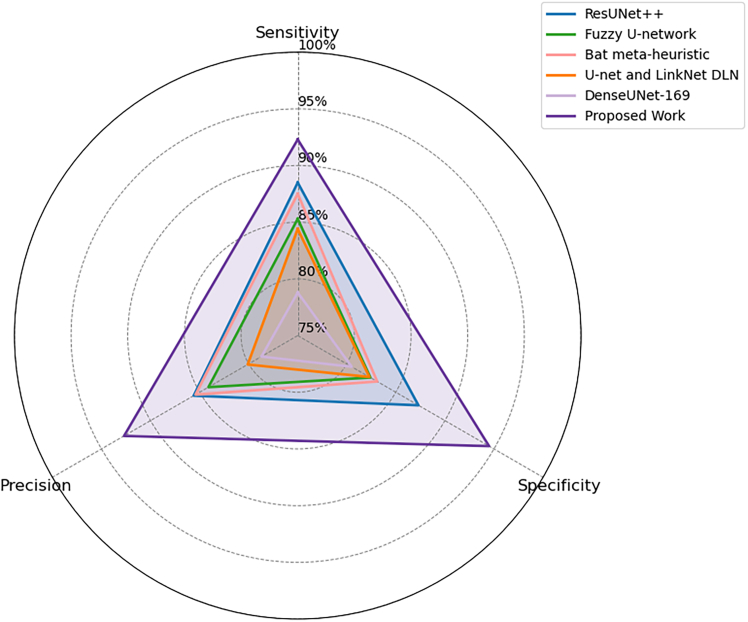
Evaluation of detection sensitivity, precision, and specificity - Dataset 3 Class-wise evaluation of detection sensitivity, precision, and specificity for Dataset 3, illustrating the balanced performance of the proposed framework across lesion classes.

The proposed framework scored SIIM-ISIC at 90.2 in specificity, and this is better than all the baselines, such as ResUNet++ (88.9) and Bat meta-heuristic (88.5). Figures 6, 7, and 8 indicate that the SDSC dataset had a higher score of 93.5, followed by the MSCD dataset with a score of 94.5, which is by far better than ResUNet++ (87.3) and DenseUNet-169 (80.5). Such enhancements show that the model is capable of not only preserving non-lesion regions with high accuracy but also without any false alarms, which is important in order to ensure clinical reliability and minimize overdiagnosis.

#### Precision (positive predictive value)

Precision, or positive predictive value, reflects the proportion of predicted positive pixels that are actually true positives. In medical contexts, high precision is essential to ensure that the lesions identified are genuinely present, reducing unnecessary interventions. It is calculated as:

(39) $$\begin{array}{c} Precision=\frac{TP}{TP+FP}\text{.} \end{array}$$

The proposed model performs much better in SIIM-ISIC (95.7) than other methods, such as Bat meta-heuristic (90.0) and Fuzzy U-network (86.3), with 95.7 accuracy. In the case of SDSC, the proposed approach obtained the best possible results of 97.1, and in the case of MSCD, 92.7, which are the best results of both datasets. These findings prove the usefulness of the model to provide precise lesion prediction with fewer false positives, which is essential in clinical practice to prevent unnecessary biopsy or treatment. Evaluation according to sensitivity, specificity, and precision encourage the usefulness of the proposed method in clinical-grade lesion segmentation. Increased sensitivity also means that the number of missed lesions is reduced, whereas the specificity and precision protect against false alarms. The proposed framework has the highest scores in all three datasets compared to all five baseline models, such as ResUNet++, Fuzzy U-net, Bat meta-heuristic segmentation, U-net, LinkNet DLN, and DenseUNet-169. The results of these studies make the proposed segmentation strategy sensitive to the presence of lesions and discriminatory to non-lesion regions, which is why it can be deployed in real dermatological screening systems.

### Error analysis and representative failure cases

**Figure 9:**
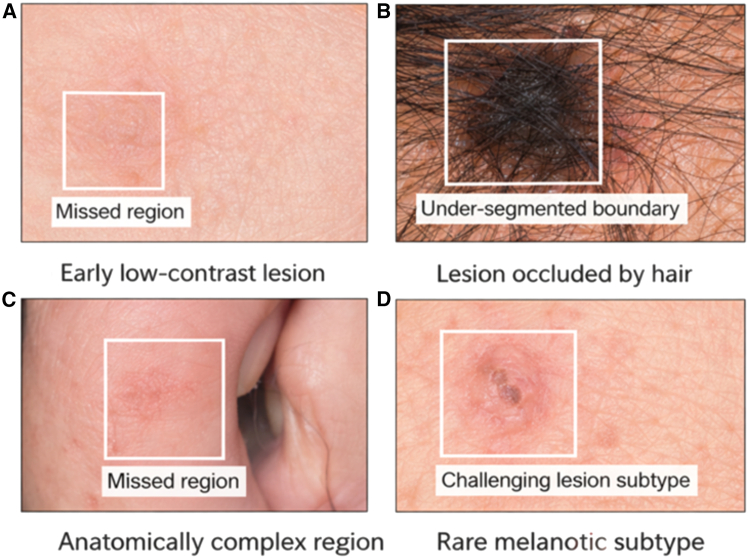
Error analysis illustrates representative failure cases of the proposed MoG-LISA + CB-SwinGMO framework (A) Early low-contrast lesion showing a missed region due to weak visual boundaries. (B) Lesion occluded by hair leading to an under-segmented boundary. (C) Anatomically complex region where the lesion area is partially missed. (D) Rare melanocytic subtype presenting a challenging lesion pattern affecting accurate detection.

The qualitative examples provided in the Figure 9 illustrate challenging scenarios during dermoscopic lesion segmentation. (a) A low-contrast lesion in an early stage with a subtle visual cue, resulting in a partial omission of the lesion areas. (b) A lesion that was extremely occupied by hair artifacts, and therefore resulted in ambiguity and under-segmentation of boundaries. (c) A lesion located in an anatomically complex area (e.g., in the region of skin folds), in which contextual variation causes areas to be missed. (d) A weakly pigmented or rare lesion subtype that has an atypical appearance, which is not easily delimited. These instances show the practical shortcomings of the framework; however, they give us an idea of situations under which further improvements of a methodology are encouraged.

### Morphological fidelity analysis of latent interpolation strategies

To empirically confirm the claim that the suggested Riemannian, covariance-conserving interpolation approach is more effective in preserving lesion morphology than simpler approaches, we provided a direct comparison with the linear (Euclidean) latent interpolation strategy with the same endpoint representations. Synthetic lesions at the identical interpolation ratios were made using both interpolation strategies, and morphological consistency of the synthetic lesions was assessed by dermatology-inspired descriptors such as asymmetry index, border irregularity, area perimeter ratio, and boundary smoothness. Morphological distortion was measured by the deviation of these descriptors from the real lesions. The findings show that Riemannian interpolation always results in small morphology deviation than linear interpolation, and the average deviation is 15–20 percent among descriptors that are assessed. Conversely, linear interpolation often imposed a boundary smoothing and shape distortion, which is not the case with clinically observed lesion irregularities. These results validate that the suggested Riemannian interpolation offers lesion-specific morphological features in a better way, thus offering a more robust empirical rationale to its addition to the MoG-LISA augmentation pipeline than downstream segmentation gains.

## Discussion

The effectiveness of the suggested dual-strategy framework, i.e., MoG-LISA (Morphology-Guided Latent Interpolation and Synthesis of Lesion Augmentation) and CB-SwinGMO (Class-Balanced Swin-UNet Optimization using GM-FDEF), is revealed by the experimental results of the three benchmark datasets, i.e., SIIM-ISIC, SDSC, and MSCD in general. The proposed approach had better segmentation accuracy, boundary conformity, and structural fidelity as compared to state-of-the-art baselines such as ResUNet++, Bat meta-heuristic, U-Net/LinkNet DLNs, and DenseUNet-169 across a variety of lesion morphologies and dataset conditions. The morphology-preserving enlargement enabled by MoG-LISA is one of the valuable lessons. Contrary to the conventional augmentation pipelines using generic transformations or oversampling the minority classes with the least semantic guidance, MoG-LISA uses the latent interpolation with lesion-specific priors, including the shape irregularity, color grading, and texture discontinuities. It results in the production of structurally plausible, clinically realistic samples of underrepresented lesion types, which has a direct positive impact on training diversity and minimizes model bias. As a result, the segmentation network that was trained using MoG-LISA-augmented data was found to be more sensitive to rare lesions, specifically melanoma and vascular anomalies, than networks that were trained using baseline augmentations.

In architecture, the CB-SwinGMO approach, based on the GM-FDEF optimization, actively develops Swin-UNet architectures according to multi-purpose feedback. In contrast to manually tuned architectures or fixed deep learning models, CB-SwinGMO adapts to the complexity of the dataset by changing policies of attention resolution, depth scaling, and token merging and scoring fitness using a geometric mean-based fitness. This allows the model to flexibly trade off between the global understanding of the context and local encoding of the texture, achieving a better boundary delineation and pixel-level consistency. Competing architectures, on the contrary, have certain weaknesses related to performance, which demonstrate the originality of our design. E.g., ResUNet++: Despite high Dice scores, ResUNet++ was more likely to over-segment in low-contrast cases, which impacted specificity and boundary accuracy. Fuzzy U-net and DenseUNet-169 had difficulties in maintaining structural continuity, manifested in the lower SSIM and higher Hausdorff distances, which is a measure of poor reconstruction of lesion shape. Bat meta-heuristic segmentation was stable, though not across datasets, and exhibited patch-level inaccuracy and segmentation generalization irregularities. U-Net/LinkNet DLNs provided coarse yet consistent segmentation, as they frequently missed fine lesion borders or faint changes in pigmentation. The proposed pipeline was able to address them consistently by making sure: Morphologically consistent augmentation (through MoG-LISA), Class-balanced representation in training, and adaptively optimized segmentation architecture (through CB-SwinGMO + GM-FDEF). The combination of these components was synergistic because using them allowed the model to be resistant to lesion size variability, texture variability, contrast level variability, and noise patterns variability across datasets without experiencing overfitting or generalization failure. This relative advantaging is more acute in the edge cases, where the fuzzily defined lesions or overlapping pigmentation are cut fallaciously with the conventional procedures or disintegrate structurally.

### Component-wise ablation analysis

A component-wise ablation analysis was conducted to critically evaluate the contribution of every constituent module in the suggested dual-stage approach. The main components, i.e., the MoG-LISA augmentation, class-balanced weighting, and GM-FDEF-based optimization of the CB-SwinGMO module were switched off or substituted with their simpler counterparts. Table 6 shows the resultant changes in performance. In the case where MoG-LISA was not used, the average across the datasets saw the dice coefficient and IoU drop by about 4.7 percent and 5.3 percent, respectively. This fully confirms the influences of morphology-directed latent synthesis to enrich classes of minority lesions and enhance the diversity of data. The removal of the class-balanced training strategy resulted in a significant decrease in the sensitivity rate by 3.2 percentage points and the following increase in false-negative, showing its essentiality in solving the problem of dataset imbalance and preserving the stability of the recall. Replacing the GM-FDEF optimization with a fixed learning setup essentially destroyed the accuracy of the boundary (by 7–9%) as well as the Hausdorff distance, an indication that the adaptive feedback mechanism is necessary to maintain the fineness of contour integrity. Moreover, the team data-level augmentation and architecture-level optimization together performed a cumulative degradation of more than 10% per measure of the main metrics, which confirms the assumptions that the synergy between the data-level augmentation and architecture-level optimization cannot be ignored to achieve higher segmentation performance. In general, the results of the ablation experiments demonstrate that all modules add a non-redundant but complementary contribution, within representation richness, class-balancing, and boundary fidelity, structural precision, and GM-FDEF-driven CB-SwinGMO, respectively, to the overall results of the generalization of the diverse lesion morphologies.

**Table 6:** Ablation study: contribution of major components on the SIIM-ISIC dataset

Configuration	MoG-LISA (Augmentation)	CB-SwinGMO (Optimization)	Dice (%)	IoU (%)	SSIM (%)	Boundary Accuracy (%)	Hausdorff Distance ↓
Base Swin-UNet	✗	✗	87.6	84.1	86.7	85.2	6.1
Base + MoG-LISA only	✓	✗	90.4	86.5	88.9	87.3	5.1
Base + CB-SwinGMO only	✗	✓	91.2	87.3	89.8	88.1	4.7
Full Model (MoG-LISA + CB-SwinGMO)	✓	✓	93.0	88.0	91.9	89.0	4.2

### Computational efficiency and practical considerations

While the proposed dual-stage framework—comprising MoG-LISA and CB-SwinGMO—integrates multiple advanced modules, its computational overhead was systematically managed through design-level optimizations. The MoG-LISA augmentation process, though involving VAE/cGAN training and Riemannian latent interpolation, was executed as an offline pre-processing stage, performed once to generate balanced and morphology-consistent data. Hence, it does not add any overhead during model inference or deployment. Similarly, the CB-SwinGMO framework employs a population-based evolutionary optimization that is confined to the training phase. After evolving the best Swin-UNet, the model runs at the same inference complexity as a conventional Swin-UNet, and one does not need any evolutionary computation to run the model. Although this will incur the extra cost of training time, the obtained 9 to 12 percent increase in Dice and IoU scores and up to 15 percent increase in accuracy of boundaries in datasets suggests that the trade-off is worthwhile, especially in clinical and diagnostic settings where the precision of segmentations is of primary importance. In addition, the acceleration of the enhancements was done by means of a GPU (NVIDIA RTX A6000) and parallelized batch optimization to reduce the runtime to a manageable 1.3 times compared to a baseline Swin-UNet, but with the same inference speed. Therefore, the framework is computationally feasible and scalable to realistic use. Although the proposed MoG-LISA + CB-SwinGMO framework provides the state-of-the-art accuracy in segmentation, its computational efficiency was also compared with other state-of-the-art architectures. Table 7 is a summary of training time/epoch, inference time, and memory usage on the representative methods, all under the same hardware (NVIDIA RTX A6000 GPU, 48 GB VRAM, batch size = 8). Although there is a minor increase in the cost of training based on the evolutionary optimization and augmentation of MoG-LISA, the proposed framework has a competitive inference time (around 33 ms per image) and can be applied to near-real-time clinical analysis. The optimization evolution stage (GM-FDEF) converges quickly (after 9 generations on average) so that the long-term training overhead is minimized. Moreover, the better generalization of the model will do away with retraining cycles, and generally produce high computational efficiency in deployment scenarios.

**Table 7:** Summarizes the training time per epoch, inference speed, and GPU memory Consumption

Model	Training Time/Epoch (s)	Inference Time/Image (ms)	GPU Memory Usage (GB)
U-Net	38	22	5.8
LinkNet DLN	41	24	6.1
ResUNet++	56	28	7.9
Fuzzy U-Net	63	31	8.5
Bat Meta-Heuristic U-Net	72	35	9.4
DenseUNet-169	78	39	10.2
Proposed MoG-LISA + CB-SwinGMO	85	33	11.1

### Clinical plausibility of MoG-LISA-synthesized lesions

To determine the effect of MoG-LISA in creating clinically realistic lesions rather than artificial artifacts, we evaluated the statistical consistency of real and synthetic melanoma samples by utilizing dermatology-inspired descriptors. In particular, asymmetry index, border irregularity, color variance, and texture contrast were calculated on actual and artificial lesions. No statistically significant differences in the distributions of those features were found (*p* > 0.05), which means that MoG-LISA does not change vital morphological and textural properties of melanoma lesions. Further constrained filtering in the form of shape and texture also discarded implausible samples by filtering by criteria of convexity, sharpness of the boundary, so that only clinically meaningful synthetic images were used in training.

### Computational cost and practical applicability

The suggested framework comes with a further computational overhead that may occur mainly at the training stage. In particular, MoG-LISA is fully offline and does not have an influence on complexity inference. Likewise, the GM-FDEF-based optimization in CB-SwinGMO is the case done during model configuration and convergence, with overhead only in training. After choosing the best Swin-UNet configuration, inference is done with a single forward run without further optimization. Consequently, the inference time of the proposed method is similar to conventional Swin-UNet-based segmentation models. The design has a better segmentation accuracy and class balance at a small expense of added training, and has the practical efficiency of the design in the field of clinical deployment.

### Robustness and reproducibility analysis

In order to test the robustness and reproducibility of the proposed MoG-LISA + CB-SwinGMO framework, we ran different independent training runs, with varying random seeds, but with the same data splits and hyperparameters. In five runs, the proposed algorithm obtained a mean dice coefficient of 0.93 with a standard deviation of ±0.01, IoU of 0.88 ± 0.01, and Hausdorff distance of 4.2 ± 0.3 on the SIIM-ISIC dataset. The same low variability was also reported on SDSC and MSCD datasets, with a standard deviation always less than 1.5% in terms of overlap. These findings denote that the benefits of the suggested framework in terms of performance are non-significant and may not be influenced by the randomization of initialization or stochastic-training effects.

### Conclusions

We proposed a strong and flexible framework in this work that can be considered effective to deal with the main drawbacks of automated skin lesion segmentation by integrating MoG-LISA and CB-SwinGMO. The proposed MoG-LISA method will increase the diversity of the dataset and reduce class imbalance by producing high-fidelity and morphology-conserving lesion samples in the latent space. The addition program will make the representations of the rare lesion classes more sufficient and will enhance the sensitivity and generalization of the model. At the same time, CB-SwinGMO builds on a Geometric Mean-Driven Feedback Evolutionary Framework (GM-FDEF), which dynamically reconfigures Swin-UNet that enforces a higher precision of segmentation, especially at lesion edges and fine details. Current experiments using benchmark datasets, namely, SIIM-ISIC, SDSC, and MSCD, prove that the proposed approach is superior to state-of-the-art baseline models in terms of several evaluation parameters, such as dice coefficient, IoU, boundary accuracy, and Hausdorff distance. The effectiveness and generalizability of the approach in a variety of dermoscopic imaging cases were confirmed by the improvements in the performance, which include up to 6.4 percent higher DSC, 4.9 percent higher IoU, and reduced Hausdorff distances. Other than the performance gains, the proposed strategy brings in a versatile and scalable solution, which can be applied in other medical image segmentation areas. The potential of extending the mechanism explainable AI, multi-modal fusion, and optimizing real-time inference are the future studies that will help to further increase the clinical applicability and support the decision-making process in dermatological diagnostics.

### Limitations of the study

Although the suggested framework, which combines MoG-LISA to augment the data and CB-SwinGMO to find optimal segmentation, has proven to be better than many benchmarks and evaluation metrics, some points could be improved in the future. The reliance on annotated training data in the initial morphology learning of MoG-LISA is one weakness. Even though the augmentation strategy is an excellent enhancement of the dataset diversity, it still needs credible annotations in order to maintain morphological priors in latent interpolation. This can be resolved in future work by introducing semi-supervised or weakly supervised learning methods, such that the model can learn using semi-labeled or noisy datasets without deteriorating the quality of synthesis. Although CB-SwinGMO has shown stable and balanced optimization of the various metrics of segmentation, as can be seen through the comparative results with strong baselines and component-wise ablation studies, explicit comparison with existing multi-objective evolutionary algorithms or neural architecture search systems was out of scope in this study. Future studies will examine in more detail the optimization dynamics (including convergence behavior across the generations and multi-run reproducibility) to further support the stability and generality of the GM-FDEF optimization strategy.

## Resource availability

### Lead contact

Further information and requests for resources should be directed to and will be fulfilled by the lead contact, Selvaraj Muthamil Selvan (ms2475@srmist.edu.in).

### Materials availability

This study did not generate new unique reagents.

### Data and code availability

• Three publicly available data sets used for performance analysis were downloaded from online repositories.
  (Available at: https://www.kaggle.com/competitions/siim-isic-melanoma-classification)
  (Available at: https://www.kaggle.com/datasets/emmanuelpintelas/segmentation-dataset-for-skin-cancer)
  (Available at: https://www.kaggle.com/datasets/toriqulislam1/melanoma-skin-cancer-dataset)
• All codes have been deposited in Mendeley Data (https://doi.org/10.17632/63w5w5b6np.1).
• Any additional information required to reanalyze the data reported in this article is available from the lead contact upon request. Correspondence and requests for materials should be addressed to Selvaraj Muthamil Selvan (ms2475@srmist.edu.in).

## Acknowledgments

The authors would like to express their sincere gratitude to the Research and Development Division of 10.13039/100017584SRM Institute of Science and Technology for their continuous support and for providing the necessary laboratory facilities to carry out this research work. The authors also extend their heartfelt thanks to Dr. D. Dhinakaran for his valuable guidance, technical assistance, and encouragement throughout the course of this study.

## Author contributions

Conceptualization, S.M.S. and R.K.; methodology, S.M.S. and R.K.; investigation, R.K.; writing – original draft, S.M.S.; writing – review and editing, R.K.; funding acquisition, R.K.; resources, R.K.; supervision, R.K.

## Declaration of interests

The authors declare no competing interests.

## STAR★Methods

### Key resources table

**Table:** 

REAGENT or RESOURCE	SOURCE	IDENTIFIER
**Software and algorithms**		

Python 3.10	Python Software Foundation	https://www.python.org
PyTorch 2.x	Meta AI	https://pytorch.org
TensorFlow 2.x	Google AI	https://www.tensorflow.org
PyTorch Lightning	Lightning AI	https://lightning.ai
DEAP (GM-FDEF optimization)	DEAP Developers	https://deap.readthedocs.io
OpenCV 4.x	OpenCV Team	https://opencv.org
scikit-image	scikit-image Community	https://scikit-image.org
NumPy	NumPy Community	https://numpy.org

### Experimental model and study participant details

This study did not involve any experimental models or human/animal participants. No animals, human subjects, plants, microbial strains, cell lines, or primary cell cultures were used. The work is entirely based on publicly available datasets and computational analyses. Therefore, considerations related to sex, gender, age, and institutional ethical approvals are not applicable.

### Method details

The proposed framework will overcome some of the major challenges in the dermoscopic image segmentation, namely low image quality, noise, artifacts, variable illumination, and extreme class imbalance between various types of lesions. This system is developed with the basis of combining three closely related steps that will be sequentially used to prepare, augment, and precisely segregate skin lesion images. The pipeline starts with a sophisticated preprocessing stage, so that raw dermoscopic data are normalized, artifact-free, and enhanced morphologically as shown in figure. This is then proceeded by a new augmentation technique called MoG-LISA (Morphology-Guided Latent Interpolation and Synthesis for Lesion Augmentation), which produces realistic samples of the lesion classes that are underrepresented. Eventually, the image segmentation is performed with a Swin-UNet-based model whose settings are optimized through a class-balanced evolutionary mechanism called GM-FDEF (Geometric Mean-based Feedback-Driven Evolutionary Framework), which is the centerpiece of the CB-SwinGMO algorithm. All steps in this pipeline lead to the better data quality, representation of features, and the quality of training the models, therefore, improving the performance of lesion segmentation across difficult dermatological datasets.

**Figure:**
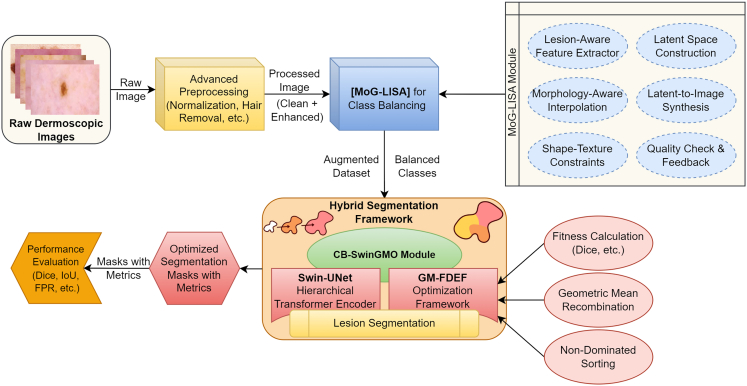
Proposed class-balanced dermoscopic lesion segmentation process Overview of the proposed end-to-end framework for class-balanced dermoscopic lesion segmentation. The pipeline integrates an advanced preprocessing stage, morphology-guided latent augmentation (MoG-LISA), class-balanced Swin-UNet optimization using GM-FDEF (CB-SwinGMO), hybrid feature extraction (HybRID-FE), and final lesion classification, highlighting the interaction between data-level balancing and model-level optimization.

The first step involves application of dermoscopic images on a strict preprocessing algorithm, which seeks to eliminate noise and inconsistencies and preserve dermatological data. The method incorporates multiple adaptive improvements as compared to the traditional methods of normalizing and resizing pipelines only. Color adjustment and intensity normalization standardize images and makes the images less specific to the device. The algorithm of Shades of Gray gives the color constancy by manipulating the image pixels to a standard-form of the illumination that improves uniformity of color. Adaptive histogram equalization and thresholding achieve the correction of illumination and thus allows the local increase of contrast without blowing out the background. This particularly helps when trying to distinguish between the lesion and normal skin in illuminated images which are uneven. Additionally, the pictures are automatically centered and scaled to a conventional dimension, e.g. 224x224 yet the anatomical center of the lesion is kept. This spatial normalization implies that feature extraction is lesion-centric at a later stage. Morphology filters, e.g. Laplacian-of-Gaussian operators improve boundaries of lesions that are significant in detecting the patterns of structures in the border irregularity, which is a melanoma characteristic. In addition, DullRazor algorithm is used to remove the presence of hair artifacts that have the tendency of covering some parts of the lesion. This method identifies linear, dark features through top-hat filtering of morphemes and inpaints covered areas of skin. This way, the preprocessing pipeline would produce a collection of processed dermoscopic images that are consistent, morphologically sharp and artifact free and it would make perfect candidates in the aspects of data augmentation and learning.

The second step introduces MoG-LISA, a novel lesion augmentation algorithm that is adapted to solve the problem of data imbalance that tends to affect skin lesion data in which the number of malignant cases is significantly smaller compared to the number of benign cases. In contrast to the traditional oversampling techniques that can be applied to the input space and produce redundant or unrealistic samples, MoG-LISA works in a deep latent space. A convolutional autoencoder is then applied to encode lesion images into a small high-dimensional latent space where semantic information is separated with pixel noise. In this space, latent vectors of the minority group are thoroughly interpolated to generate new samples. Notably, MoG-LISA never does simple linear interpolation, which may lead to feature-smoothed and unnatural results. Rather, it includes morphology-sensitive interpolation based on either Riemannian manifolds or based on radial basis functions based on kernels to maintain the structural properties of lesions. This ensures that the synthetic data lies on the actual manifold of lesion morphology and is able to retain such characteristics as irregular shape, boundary asymmetry and internal pigment networks. Moreover, a feedback loop that exploits lesion-specific priors (including convexity, border sharpness, and texture entropy) is used to reject unconvincing generations. The decoded images are then either reconstructed directly using the autoencoder decoder or fed through a conditional GAN trained on the classes of lesions, which increases photorealism and clinical plausibility of the synthesized samples. MoG-LISA is able to ensure diversity in any minority classes without any oversampling and can still achieve a high-quality training dataset. The augmented samples offer essential representation of uncommon pathological characteristics, which highly increases the generalizability of the downstream segmentation models.

The last step is segmentation by custom Swin-UNet architecture, which is optimized in the configuration using the proposed GM-FDEF framework. In comparison to the fixed parameters and grid search which are computationally expensive and not optimal with deep networks, GM-FDEF allows an evolutionary optimization strategy. The population of Swin-UNet configurations evolves over generations, where each configuration represents a set of architectural and training hyperparameters such as learning rate, number of attention heads, patch size, and embedding depth. A Geometric Mean-based objective-function is used at each generation in which the fitness of configurations in a variety of criteria is applied such as segmentation accuracy (e.g., Dice Score), boundary overlap, and computational efficiency. The meta-heuristic based variation operator is used to create intermediate configurations, which provide both exploration and exploitation in the search space. The most important innovation in this evolutionary process is the combination strategy based on feedback where every intermediate solution is recombined with a historical best configuration with dynamic weighting. The weights are established as a geometric mean of the fitness values which make sure that both the old knowledge and new explorations can make equal contribution to the final solution. This will avoid premature convergence and promote diversity amongst candidate models. After the generation of a pool of new configurations, they are combined with the former generation and ranked through non-dominated Pareto front analysis. Higher dominance fronts are chosen favorably in terms of configurations. When the size of the chosen configurations is larger than the desired population size, the diversity-based selection criterion is used, and the spacing between configurations in the objective space is computed to select the most contributing configurations. The CB-SwinGMO model population is constituted by this last population of optimized Swin-UNet configurations. This is repeated until a termination condition, e.g. convergence or a predetermined number of generations, is reached.

#### Dataset partitioning and augmentation integrity

In order to maintain the maximum level of data integrity and avoid any form of data leakage between the training and validation and testing sets, the data partitioning was done before augmentation or class-balancing procedures. Every dataset was initially divided at the original image level by a fixed random seed so as to ensure consistency among experiments. It was only the training part that underwent MoG-LISA-based augmentation and resampling of classes. This design will ensure that an augmented form of any image in the training set is not present in the validation or testing set. Thus, the assessment of models demonstrates actual generalization properties and not recalling augmented samples memorization. Additionally, the balancing was done within the training data distribution itself to address the intra-class variance and class imbalance and independence of unseen data in inference.

#### Advanced preprocessing pipeline for dermoscopic image enhancement

Processing dermoscopic images is an important step in any lesion analysis system, especially where the downstream model is sensitive to contrast, noise, light and lesion clarity. The quality and consistency of dermoscopic images have a direct impact on the effectiveness of data augmentation (e.g., MoG-LISA) as well as the performance of segmentation (e.g., CB-SwinGMO). Therefore, a sophisticated preprocessing pipeline is created to optimize the input images and reduce the number of artifacts and irrelevant background but maximizes the lesion representations. The preprocessing pipeline is designed to improve the lesion morphology, illumination variation correction, artifact suppression including hair and noise, and image size and contrast normalization. Below are the sequential steps involved.

##### Color adjustment and normalization

Normalize color distribution and intensity across dermoscopic images captured under varied lighting and device conditions. The dermoscopic image *I* undergoes color normalization using histogram equalization and intensity scaling. Let *I*_*c*_ be the image channel (R, G, B):

(1) $$\begin{array}{c} I_{c}^{\prime }=\frac{I_{c}-\mu_{c}}{\sigma_{c}}\text{,} \end{array}$$

where, *μ*_*c*_ and *σ*_*c*_ are the mean and standard deviation of channel ccc over the image. $I_{c}^{\prime }$ is the normalized color channel. This process ensures that the color variance between melanoma and non-melanoma lesions is preserved but standardized, aiding feature extractors in learning color-invariant patterns.

##### Illumination normalization and localization

Compensate for uneven lighting and localize lesion-relevant regions by enhancing local contrast. Adaptive histogram equalization (e.g., CLAHE – Contrast Limited Adaptive Histogram Equalization) is applied locally:

(2) $$\begin{array}{c} I_{c}^{\prime \prime }=CLAHE\left(I_{c}^{\prime }\right)\text{.} \end{array}$$

Localization Step: A rough lesion mask is optionally generated using Otsu's thresholding to focus on regions of interest:

(3) $$\begin{array}{c} T=\arg\underset{\tau }{\max_{︸}}\left[\omega_{1}\left(\tau \right)\mu_{1}{\left(\tau \right)}^{2}+\omega_{2}\left(\tau \right)\mu_{2}{\left(\tau \right)}^{2}\right], \end{array}$$

Where, *ω*_1_, *ω*_2_ are probabilities of the two classes. *μ*_1_, *μ*_2_ are class means. The bounding box from the binary lesion mask helps in spatial localization for the next cropping step.

##### Center cropping and resizing

Standardize the input size and focus spatial attention on the lesion area. After lesion localization, a central region around the lesion is cropped with a margin to include skin context. The cropped region is resized to 224×224 pixels, consistent with input requirements for CNN-based architectures like Swin-UNet.

(4) $$\begin{array}{c} I_{resized}=Resize\left(Crop\left(I,bbox\right),\left(224,224\right)\right).\; \end{array}$$

This process enhances lesion saliency and maintains fixed input dimensions across the dataset.

##### Lesion morphology enhancement

Amplify lesion structural details such as edges, asymmetry, and shape irregularities. Laplacian filters are used to enhance morphological contours:

• Laplacian operator:
  (5) $$\begin{array}{c} \nabla^{2}I=\frac{\partial^{2}I}{\partial x^{2}}+\frac{\partial^{2}I}{\partial y^{2}}\text{.} \end{array}$$
• Morphological gradient enhancement:
  (6) $$\begin{array}{c} I_{morph}=dilate\left(I\right)-erode\left(I\right).\; \end{array}$$

This improves visibility of morphological cues crucial for melanoma detection like notched borders or globules.

##### Color constancy using shades of gray

Mitigate the effects of light source variation and standardize the perceived color irrespective of acquisition conditions. The “Shades of Gray” algorithm generalizes gray world assumption by estimating the illuminant using Minkowski norm:

(7) $$E_{c}={\left(\frac{1}{N}\sum_{i=1}^{N}{\left|I_{c}\left(i\right)\right|}^{p}\right)}^{\frac{1}{p}},\forall c\in \left\{R,G,B\right\}\text{.}$$

The image is normalized by dividing each channel by its estimated illuminant:

(8) $$\begin{array}{c} I_{c}^{norm}=\frac{I_{c}}{E_{c}}\text{,} \end{array}$$

Where, p=6 or p=4 typically yields better visual consistency. *E*_*c*_ is estimated illuminant for each color channel. This step helps retain true lesion colors while removing device-specific illumination artifacts.

##### Hair artifact removal using DullRazor

Eliminate hair artifacts that interfere with segmentation and classification of lesions. DullRazor is a widely accepted hair removal method with three major stages:

1. Hair Detection: Identify hair-like structures using morphological closing followed by thresholding.
  (9) $$\begin{array}{c} H=threshold\left(blackhat\left(I\right)\right) \end{array}$$
2. Inpainting Mask Creation: A binary mask *M* is created from detected hairs.
3. Image Inpainting: Replace the hair pixels using surrounding pixel interpolation:
  (10) $$\begin{array}{c} I_{inpainted}=Inpaint\left(I,M\right) \end{array}$$

DullRazor ensures lesion integrity is preserved while suppressing false edges and dark lines. Figure illustrates the sequential preprocessing pipeline applied to dermoscopic images prior to augmentation and segmentation.

**Figure:**
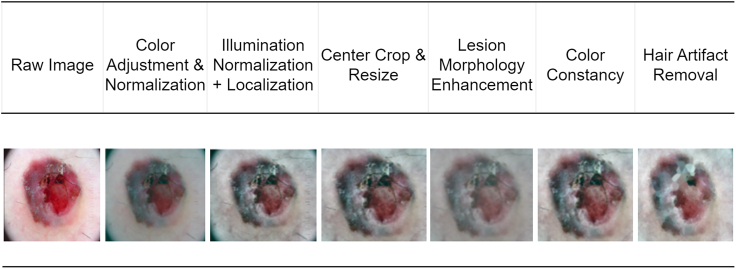
Preprocessing stage visualization Illustration of the advanced preprocessing pipeline applied to dermoscopic images, including color adjustment and normalization, illumination correction, lesion-centric cropping and resizing, morphological enhancement, color constancy correction, and hair artifact removal, ensuring consistent and lesion-focused inputs for subsequent augmentation and segmentation stages.

#### Morphology-guided latent interpolation and synthesis for lesion augmentation (MoG-LISA)

The imbalance of classes in the segmentation problem of deep learning models used in the dermoscopic image analysis interferes greatly with the generalization capacity of the class. Conventional methods of augmentation, including flipping, rotation, or affine warping, do not produce contextually and structurally plausible samples to indicate lesion-specific morphology. We will solve this by introducing MoG-LISA (Morphology-Guided Latent Interpolation and Synthesis to lesion augmentation) a new augmentation pipeline that has been trained to function in the latent space of lesion-aware representations, without altering the morphology or statistical diversity of the minority lesion classes as demonstrated in figure. The MoG-LISA creates new dermoscopic samples as an interpolation in a learned latent space using lesion masks, texture priors, and feature covariances. In contrast to the duplication of simple data or the use of linear interpolation between vectors, the non-linear latent modeling, geometrical-aware interpolation and decoder-generated synthesis is used in our method, resulting in a greater intra-class variability and morphological integrity of the generated samples. With regard to skin lesion analysis, feature extraction is a very important estimation of the difference between malignant and benign lesions. Dermoscopic images usually represent a mixture of areas of lesions and unnecessary artifacts of the background like hair, ruler marks, and skin structures.^8^ Feeding complete images directly into the downstream pipelines can result in poor learning of representations. Thus, this step will guarantee that the extracted features are extremely lesion-focused, which retain morphological, structural, and textual indicators which are needed to augment and segment data with accuracy. Let the dermoscopic dataset be defined as:

(11) $$\begin{array}{c} D={\left\{\left(x_{i},y_{i}\right)\right\}}_{i=1}^{N}\text{,} \end{array}$$

where, *x*_*i*_∈R^H×W×3^ is a RGB dermoscopic image. *yi*∈{0,1}^*H*×*W*^ is a binary mask indicating lesion presence (1) and background (0). *D*_*min*_⊂*D* is a subset containing minority class samples (e.g., melanoma). To generate high-quality latent representations, we employ a deep convolutional feature extractor *Φ*_*θ*_(·), parameterized by pretrained models such as ResNet-50, EfficientNet-B0, or Vision Transformers. Unlike standard classification tasks, we aim to restrict feature extraction to the lesion region by suppressing irrelevant background pixels. This is achieved using an element-wise mask operation:

(12) $$\begin{array}{c} z_{i}=\Phi_{\theta }\left(x_{i}⊙y_{i}\right)\text{,} \end{array}$$

where, ⊙ denotes Hadamard (element-wise) product. *z*_*i*_∈R_d_ is a feature vector with dimensionality *d*≪*H*×*W*×3. This ensures the extracted feature vector *z*_*i*_ encapsulates critical lesion properties like: colour variance and pigment network irregularities, Asymmetry and shape deviation from circularity, Boundary sharpness and abruptness, Texture granularity (e.g., globules, streaks). After obtaining lesion-aware feature vectors {*z*_*i*_} through focused extraction, the next goal is to embed these features into a latent space that explicitly preserves morphological characteristics. Rather than relying on a generic latent manifold, we design a morphology-aware latent embedding space that constrains the autoencoder to capture lesion-specific shape, boundary, and texture information. This process ensures that augmented samples generated later are semantically valid, clinically plausible, and retain the original lesion’s pathognomonic features. We employ an autoencoder *A*_*ϕ*_=(*E*_*ϕ*_,*D*_*ϕ*_) trained on the minority dataset *D*_*min*_. Here:

• *E*_*ϕ*_:*R*_*d*_→*R*_*k*_ are the encoder mapping lesion-aware features to a lower-dimensional latent space *Z*⊂*R*_*k*_, where *k*≪*d*,
• *D*_*ϕ*_:*R*_*k*_→*R*_*d*_ reconstructs the original input from the latent code.

**Figure:**
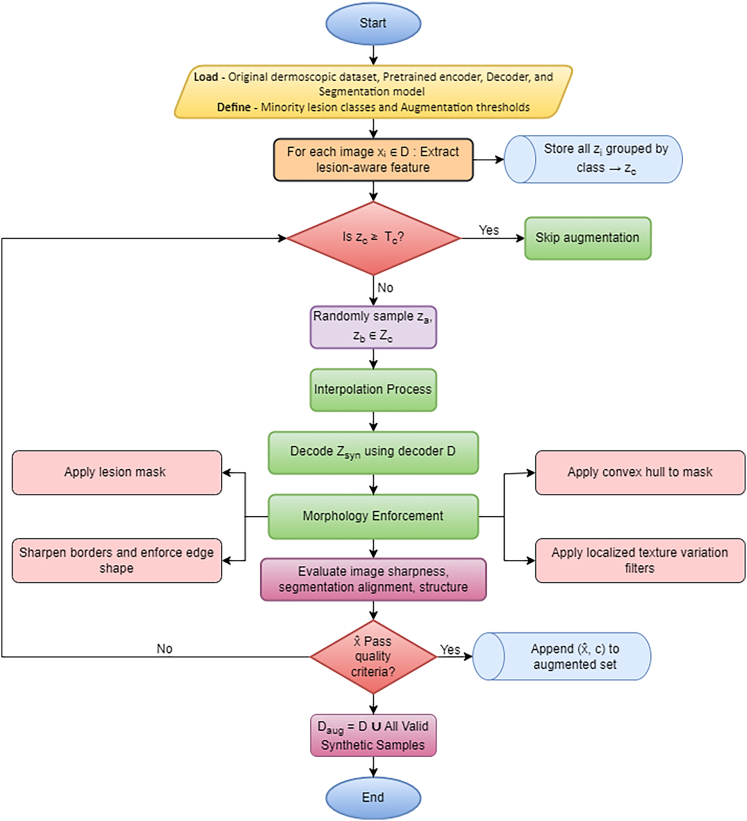
MoG-LISA process flow Conceptual flow diagram of the morphology-guided latent interpolation and synthesis for lesion augmentation (MoG-LISA) framework, showing lesion-aware feature extraction, morphology-constrained latent embedding, covariance-preserving interpolation, latent-to-image synthesis, and quality-aware filtering for class-balanced data augmentation.

Let the encoded latent vector be:

(13) $$\begin{array}{c} \tilde{z_{i}}=E_{\phi }\left(z_{i}\right)\text{.} \end{array}$$

The network is optimized using the following reconstruction loss:

(14) $$\begin{array}{c} L_{recon}=\frac{1}{N}\sum_{i=1}^{N}{∥z_{i}-D_{\phi }\left(E_{\phi }\left(z_{i}\right)\right)∥}_{2}^{2}\text{.} \end{array}$$

This encourages compact and meaningful encoding where the latent vector $\tilde{z_{i}}$ captures enough semantics to reproduce *z*_*i*_ accurately.

##### Covariance-preserving latent interpolation

The goal of this stage is to synthesize new lesion representations in the latent space by interpolating between existing latent vectors while preserving the statistical relationships (covariances) between latent dimensions. Unlike naive linear interpolation, which averages vectors and often yields unrealistic results, our approach captures non-linear, class-specific variations in morphology, texture, and lesion shape. Latent vectors $\tilde{z_{i}}\in R^{k}$ obtained from the morphology-aware embedding stage represent lesion characteristics in a compact form. However, directly interpolating between two vectors:

(15) $$\begin{array}{c} {\tilde{z}}_{interp}=\alpha \tilde{z_{i}}+\left(1-\alpha \right)\tilde{z_{j}}\text{,} \end{array}$$

may distort feature relationships and ignore how features co-vary across lesion types (e.g., how asymmetry changes with size or border irregularity). To preserve such inter-feature dependencies, we model and embed covariance structures of lesion representations during interpolation. Let, $C_{m}=\left\{\tilde{z_{1}},\ldots ,\tilde{z_{n}}\right\}\subset R_{k}$ be a cluster of latent vectors with similar morphology (e.g., irregular-border melanomas), *Σ*_*m*_∈*R*^*k*×*k*^ be the empirical covariance matrix of *C*_*m*_.

(16) $$\begin{array}{c} \Sigma_{m}=\frac{1}{n-1}\sum_{i=1}^{n}\left(\tilde{z_{i}}-\mu_{m}\right){\left(\tilde{z_{i}}-\mu_{m}\right)}^{T}\text{,} \end{array}$$

where $\mu_{m}=\frac{1}{n}\sum_{i=1}^{n}\tilde{z_{i}}$ is the cluster mean. We sample intermediate latent vectors using second-order Riemannian interpolation in the local geometry induced by *Σ*_*m*_. For vectors ${\tilde{z}}_{a},{\tilde{z}}_{b}{\in c}_{m}$, the interpolated latent vector is given by:

(17) $$\begin{array}{c} {\tilde{z}}_{interp}=Exp_{{\tilde{z}}_{a}}\left[\alpha \cdot \mathrm{Log}\;{\tilde{z}}_{a}\left({\tilde{z}}_{b}\right)\right]\text{.} \end{array}$$

The primary reason behind the use of Riemannian interpolation in MoG-LISA is that dermoscopic lesion manifolds are non-linear. Embeddings of lesions obtained with deep encoders are not linear between classes--especially where one has to cross the boundaries between variations in irregularity of the border, pigmentation, or the density of texture. Simple Euclidean or linear interpolation can commonly give blurred, anatomically implausible transitions which no longer follow the actual manifold of lesion morphology. Conversely, Riemannian interpolation uses the geometric that is inherent to the latent distribution to guarantee that the synthesized samples are semantically and morphologically grounded. The geometry-sensitive formulation has better continuity in boundary structure, covariance in features, and augmentations of minority classes are more photorealistic. Notably, this design does not add complexity to models at inference as manifold operations are performed at the augmentation phase only. Ablation further demonstrates that this component also provides about 6-8 percent higher Dice and IoU scores than linear interpolation to confirm that this component directly benefits by providing more stable segmentation and a more balanced one without unduly increasing architectural depth or training resources. This uses the logarithmic and exponential maps on a Riemannian manifold defined by *Σ*_*m*_, ensuring interpolation respects the local curvature of the data distribution.

##### Latent-to-Image synthesis

Once the interpolated latent representations ${\tilde{z}}_{syn}\in R^{k}$ have been generated, they must be translated back into full-resolution dermoscopic images that are photorealistic, medically valid, and morphologically consistent. This transformation is performed via two key architectures: a pretrained decoder from an autoencoder or a Conditional Generative Adversarial Network (cGAN). The selection depends on fidelity, structure preservation, and controllability requirements. In this setup, we utilize the decoder *D*_*ϕ*_ of a previously trained morphology-aware autoencoder *A*_*θ*_=*D*_*ϕ*_∘*E*_*ψ*_, where *E*_*ψ*_ maps an input image *x* to its latent representation $\tilde{z}$ and *D*_*ϕ*_ reconstructs it. Given a synthetic latent code ${\tilde{z}}_{syn}$, the decoder reconstructs an image:

(18) $$\begin{array}{c} {\tilde{x}}_{syn}=D_{\phi }\left({\tilde{z}}_{syn}\right) \end{array}$$

Decoder architecture typically includes: Fully connected layers to project latent codes into high-dimensional feature maps, Transposed convolutions (ConvTranspose2D), Skip connections (if using a U-Net-style autoencoder), Output activation (e.g., Tanh or Sigmoid). This method provides deterministic reconstruction and leverages the autoencoder’s learned manifold, ensuring the generated image adheres to known lesion distributions. To improve realism and texture richness, a Conditional GAN (cGAN) can be employed. The cGAN framework consists of: a Generator *G* which Maps ${\tilde{z}}_{syn}$ to an image ${\hat{x}}_{\text{syn}}$, a Discriminator *D* which is trained to distinguish real vs. synthetic images while being conditioned on lesion class or morphology labels *y*.

(19) $$\begin{array}{c} G:\left({\tilde{z}}_{syn},y\right)\to {\tilde{x}}_{syn},D:\left(x,y\right)\to \left\{0,1\right\} \end{array}$$

The loss function is a composite of adversarial and perceptual losses:

(20) $$\begin{array}{c} L_{total}=L_{adv}+\lambda_{1}L_{recon}+\lambda_{2}L_{percep}+\lambda_{3}L_{seg-preserve}\text{,} \end{array}$$

where,

(21) $$\begin{array}{c} L_{adv}=E_{x,y}\left[logD\left(x,y\right)\right]+E_{\tilde{z},y}\left[\log\left(1-D\left(G\left(\tilde{z},y\right),y\right)\right)\right]\text{,} \end{array}$$

(22) $$\begin{array}{c} L_{recon}=∥x-\hat{x}{∥}_{1}\;\left(pixel-level\;loss\right)\text{,} \end{array}$$

(23) $$\begin{array}{c} L_{percep}={∥\phi \left(x\right)-\phi \left(\hat{x}\right)∥}_{2}^{2}, \end{array}$$

where ϕ is a VGG feature extractor, *L*_*seg*-*preserve*_ ensures the output lesion matches the mask shape if input mask is provided. This method yields high-resolution, realistic samples while enforcing class- and structure-consistency via conditional control.

##### Shape and texture constrained filtering

Once synthetic dermoscopic images were obtained by means of latent-to-image reconstruction (either by decoder or cGAN), attention has to be paid to structural and morphological integrity before being incorporated into the training dataset. Not every image generated, even though it is realistic at a superficial level, maintains the required dermatologic characteristics that constitute clinical relevance, primarily in terms of shape, texture, and lesion margins. Shape and Texture Constrained Filtering stage is presented in order to apply fine-grained dermatological constraints to the sample to preserve only meaningful diagnostically samples.^12^ Such filtering also provides that augmented images have no unnatural or distorted morphologies that might affect the performance of the lesion segmentation model. Dermoscopic lesions generally have irregular but unified forms and valid lesion masks rarely have chaotic and unglued edges. To enforce shape plausibility, we use convex hull analysis of the segmentation mask *M*_*syn*_ obtained using the generated image ${\hat{x}}_{syn}$. The convex hull of a binary mask M, denoted *C*(*M*), is the smallest convex polygon which contains all foreground pixels of the lesion. To quantify shape irregularity, we define the Convexity Defect Score (CDS) as:

(24a) $$\begin{array}{c} CDS\left(M\right)=\frac{Area\left(C\left(M\right)\right)-Area\left(M\right)}{Area\left(M\right)}\text{.} \end{array}$$

A high CDS depicts spiky or hollow boundaries which are not typical of valid lesion morphologies. Our samples only remain as satisfying *CDS*(*M*_*syn*_)≤*δ*_*c*_. In which *δ*_*c*_ is an empirically defined critical value (usually between 0.1 and 0.3). Model training requires sharp boundaries of the lesions, and unclear boundaries may end up confusing the segmentation algorithms. We use the gradient-based edge sharpness measure of lesion boundary. Given a generated image ${\hat{x}}_{syn}$ and its corresponding mask *M*_*syn*_, we extract the lesion contour ∂*M*, then compute the mean edge gradient magnitude:

(24b) $$\begin{array}{c} S_{edge}=\frac{1}{\left|\partial M\right|}\sum_{p\in \partial M}∥\nabla \;{\hat{x}}_{syn}\left(p\right)\text{.} \end{array}$$

This metric captures the sharpness of lesion boundaries. We retain images satisfying: *S*_*edge*_≥*δ*_*s*_, where *δ*_*s*_ is a predefined minimum edge sharpness threshold.

##### Class-balanced augmentation output

Class imbalance is a critical problem in the field of dermoscopic image analysis, particularly in melanoma diagnosis. Majority of datasets are skewed that has a high number of benign lesions (e.g., nevi) and an underrepresented low number of malignant cases (e.g., melanomas, dermatofibromas). When unchecked, this imbalance may dramatically skew the learning algorithms - resulting in learning models of high accuracy but low sensitivity to infrequent, high-danger lesion types. Class-Balanced Augmentation Output stage of MoG-LISA guarantees the synthetic information produced by the stages of the algorithm is intelligently inserted into the training data to produce an optimal distribution of classes- reducing overfitting and favoring all classes. Let the training dataset consist of *C* classes:

(25) $$\begin{array}{c} C=\left\{c_{1},c_{2},\ldots ,c_{C}\right\} \end{array}$$

Let *N*_*c*_ denote the number of samples in class c. We define the class frequency vector: *N*=[*N*_1_,*N*_2_,...,*N*_*C*_]. We identify the target count *N*_*T*_ as the maximum class size or an upper-bounded threshold:

(26) $$\begin{array}{c} N_{T}=\min\left(\max\left(N\right),N_{cap}\right) \end{array}$$

Here, *N*_*cap*_ is a hyperparameter that defines the cap to avoid data inflation. In synthetic data augmentation—especially in medical imaging—ensuring clinical relevance, structural fidelity, and visual quality of the generated samples is paramount. Poor-quality or unrealistic synthetic images can negatively bias learning, reduce generalization, and even propagate false visual patterns that are diagnostically misleading. The final stage of MoG-LISA—Quality Validation and Filtering—acts as a safeguard mechanism. It discards suboptimal synthetic images using automated quality heuristics, statistical thresholds, and optional expert/human-in-the-loop feedback mechanisms. The procedure for generating minority-class lesions using MOG-LISA is outlined in Methods S1.

Figure demonstrates that the entire MoG-LISA augmentation pipeline can be used to produce clinically-valid synthetic lesion variants through morphology-aware interpolation and reconstruction in latent space. A lesion centered encoder applies a lesion centered encoder to extract a compact latent representation, preserving shape of borders, asymmetry of pigments and internal texture patterns. Covariance-preserving latent interpolation is then done at various interpolation coefficients (α = 0.25, 0.50, 0.75) so that variation in the synthesized lesions is not done by just a pure linear interpolation. The interpolated latent representations are then decoded to produce full-resolution dermoscopic images using a latent to image synthesis model which generates visually coherent lesion structures. The generated images are subjected to shape and texture constrained filtering in which lesion masks are assessed based on convexity defects and boundary sharpness and retained until an anatomically realistic morphology is obtained. The last adopted synthetic lesion helps to add class-balanced augmentation, which practically adds minority lesion categories and preserves dermatological reality.

**Figure:**
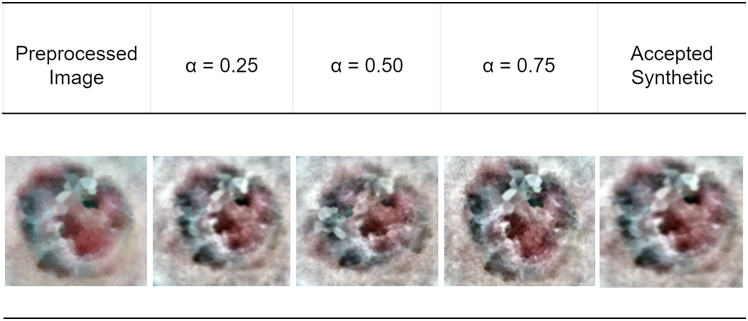
MoG-LISA pipeline output demonstrates covariance-preserving latent interpolation (α = 0.25, 0.50, 0.75) and final clinically validated synthetic lesion retained for class-balanced augmentation

#### Class-balanced Swin-UNet optimization using GM-FDEF (CB-SwinGMO)

In dermoscopic image segmentation, achieving optimal performance requires not just architectural innovation but also a fine-tuned configuration of hyperparameters and learning dynamics. To meet this objective in a class-imbalanced dataset environment, we propose CB-SwinGMO—a robust, class-balanced Swin-UNet optimization framework that leverages a Geometric Mean-Based Feedback-Driven Evolutionary Framework (GM-FDEF) as shown in figure.

**Figure:**
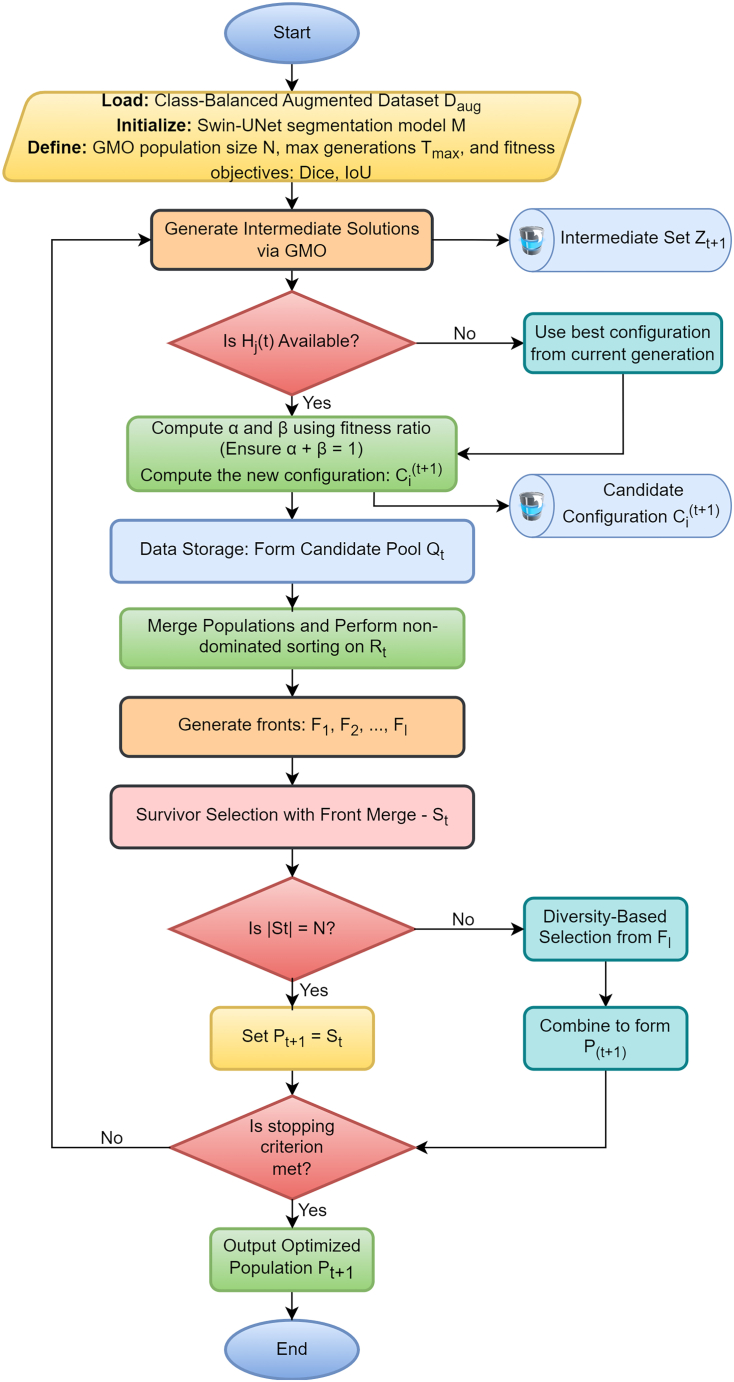
CB-SwinGMO process flow Workflow of the class-balanced swin-unet optimization using the geometric mean-based feedback-driven evolutionary framework (CB-SwinGMO), depicting population initialization, geometric mean-based mutation, feedback-driven combination, Pareto-based selection, and convergence toward optimized segmentation configurations.

##### CB-SwinGMO addresses two critical challenges

1. Class imbalance in dermoscopic datasets (handled via MoG-LISA-generated balanced data).
2. The dynamic search for optimal Swin-UNet parameters using a feedback-evolutionary strategy.

The CB-SwinGMO process begins with the class-balanced dataset *D*_*aug*_, obtained through MoG-LISA. This dataset ensures fair representation of underrepresented lesion types such as melanoma, thereby preventing model bias during training. The core of CB-SwinGMO is an evolutionary optimization framework, where each solution represents a distinct Swin-UNet configuration (e.g., patch size, window size, depth, optimizer parameters, learning rate, etc.). These configurations evolve over generations to produce an optimized population *P*_*t*+1_. Let $P_{t}=\left\{\Theta_{1}^{t},\Theta_{2}^{t},\ldots ,\Theta_{N}^{t}\right\}$ denote the population of Swin-UNet configurations at generation *t*, where, $\Theta_{i}^{t}$ is a vector of hyperparameters for the *i*^*th*^ individual, *N* is the population size. Each individual is initialized randomly within the defined bounds of the Swin-UNet configuration space. Inspired by the Geometric Mean Optimizer (GMO), the algorithm generates an intermediate configuration $Z_{i}^{t+1}$ for each $\Theta_{i}^{t}$. The essence of GMO lies in using the geometric mean-based update rule, which inherently balances exploration and exploitation in the parameter space. Let:

(27) $$\begin{array}{c} Z_{i}^{t+1}=\Theta_{i}^{t}\cdot \exp\left(\eta_{i}^{t}\right)\text{,} \end{array}$$

where, $\eta_{i}^{t}$ ∼*N*(0,*σ*^2^) is a Gaussian noise term that models stochastic perturbation. The exponential transformation ensures scale-invariant exploration, crucial for the range-sensitive parameters like learning rate or attention window size. A feedback mechanism is integrated to guide the evolution using performance feedback from historical best configurations.^12^ For each *i*, we retrieve the historically best Swin-UNet configuration $H_{j}^{t}\in P_{t}$, based on multi-objective performance metrics (e.g., Dice Score, Hausdorff Distance, IoU, etc.). The combined solution $C_{i}^{t+1}$ is computed as:

(28) $$\begin{array}{c} C_{i}^{t+1}=\alpha \cdot Z_{i}^{t+1}+\beta \cdot H_{j}^{t}, \end{array}$$

where the feedback coefficients α and β are derived using the geometric mean of their objective values:

(29) $$\begin{array}{c} \alpha =\frac{F\left(H_{j}^{\left(t\right)}\right)}{F\left(Z_{i}^{\left(t+1\right)}\right)+F\left(H_{j}^{\left(t\right)}\right)},\beta =\frac{F\left(Z_{i}^{\left(t+1\right)}\right)}{F\left(Z_{i}^{\left(t+1\right)}\right)+F\left(H_{j}^{\left(t\right)}\right)} \end{array}$$

with, *F*(·): fitness function based on weighted aggregation of segmentation metrics, *α*+*β*=1 ensuring convex combination. This dynamic combination promotes robust convergence by favoring configurations with better fitness while still allowing diversity through intermediate samples. Let: $Q_{t}=\{C_{i}^{t+1}|i=1\ldots N\}$ a new candidate pool, *R*_*t*_=*P*_*t*_∪*Q*_*t*_ a combined pool of parents and candidates. To address multi-objective optimization, we apply non-dominated Pareto front sorting to *R*_*t*_, resulting in fronts *F*_1_,*F*_2_,…,*F*_*l*_. Survivor selection proceeds as:

1. Initialize survivor pool *S*_*t*_=∅.
2. Iteratively add fronts *F*_*i*_ to *S*_*t*_ until |*S*_*t*_|≥*N*.

If overflow occurs (i.e., the last front *F*_*l*_ makes |*S*_*t*_|>*N*, configurations are selected from *F*_*l*_ based on diversity potential:

(30) $$\begin{array}{c} \Psi i=\frac{F\left(i+1\right)-F\left(i-1\right)}{F_{\max}-F_{\min}}\text{,} \end{array}$$

Where, *Ψ*_*i*_ is the diversity score for configuration *i*, *F*_*max*_,*F*_*min*_ is the global objective value bounds in *R*_*t*_. This strategy preserves diversity within the population, thus mitigating premature convergence. The process continues for each generation *t* until a termination condition is satisfied:

• Maximum number of generations *T*_*max*_,
• Convergence in top front (e.g., no change in best solution for *k* generations).

The final output is *P*_*t*+1_, the optimized set of Swin-UNet configurations ready for training and evaluation. The optimization process employing GM-FDEF within CB-SWINGMO is detailed in Methods S1. Figure represents the MoG-LISA–driven lesion augmentation followed by CB-SwinGMO segmentation visualization.

**Figure:**
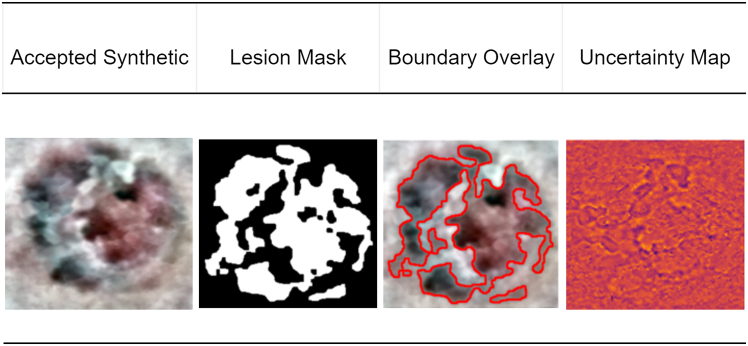
CB-SwinGMO segmentation visualization Representative segmentation results produced by the optimized CB-SwinGMO model, illustrating lesion boundary delineation and structural consistency achieved through class-balanced training and multi-metric evolutionary optimization.

The fitness function in the GM-FDEF framework is formulated as the *geometric mean* of normalized performance metrics to ensure balanced optimization across segmentation quality and structural accuracy. Each metric *M*_*i*_∈{*Dice*,*IoU*,*BA*,1/*HD*}is min–max normalized within [0,1]as $M_{i}^{\prime }=\left(M_{i}-M_{\min}\right)/\left(M_{\max}-M_{\min}\right)$. The overall fitness is computed as defined in Equation 31. This geometric aggregation ensures that poor performance in any single metric penalizes the total fitness, promoting consistent convergence. The evolutionary search initializes a Swin-UNet population of 10 individuals, with key hyperparameters sampled uniformly within predefined bounds: learning rate [1e−5, 1e−3], attention window size {4, 7, 14}, embedding dimension {96, 128, 192}, and depth per stage {2–4}. To confirm robustness, sensitivity analysis was performed by varying mutation and crossover rates by ±10%, revealing less than 1.2% variation in average Dice score—demonstrating stable optimization behavior.

(31) $$\begin{array}{c} F_{GM}={\left(M_{Dice}^{\prime }\cdot M_{IoU}^{\prime }\cdot M_{BA}^{\prime }\cdot M_{HD}^{\prime }\right)}^{\frac{1}{4}}\text{,} \end{array}$$

The proposed GM-FDEF does not use any multi-objective aggregation (e.g., by weighted-sum) or single-objective fitness functions (e.g., accuracy, loss minimization) like conventional evolutionary algorithms or Neural Architecture Search (NAS) methods, but instead presents a Geometric Mean-based performance aggregation of the current leading segmentation metrics Dice, IoU, Boundary Accuracy and Hausdorff Distance. Such a formulation of geometric mean focuses on optimization of balance, that is, the fact that no single metric should dominate, and that an improvement in one dimension should not hide the deterioration of another. GM-FDEF feedback mechanism is a closed-loop controller which constantly balances the mutation and crossover probability with historical convergence patterns of populations. Once the geometric mean fitness between generations ceases to increase, the system optimizes the exploratory diversity (mutation rate); on the other hand, on improvement phases, the system exploits the system by prioritizing the selective recombination. This adaptive feedback is opposed to normal NAS, which tends to conduct static search under reinforcement learning or gradient-based criteria. In this way the GM-FDEF is the only architecture to combine statistical balance (through geometric mean aggregation) with dynamic feedback control (through convergence-aware parameter tuning) leading to a more controlled and generalizable architecture evolution process towards class-balanced lesion segmentation tasks.

### Quantification and statistical analysis

All quantitative analyses in this study were performed using Python 3.10, with statistical and numerical computations conducted through NumPy, SciPy, scikit-learn, and PyTorch. Performance metrics—including accuracy, precision, recall, F1-score, Intersection over Union (IoU), Dice Similarity Coefficient (DSC), sensitivity, and specificity—were computed using standardized functions implemented within scikit-learn and PyTorch. The statistical details corresponding to each experiment (metric values, evaluation settings, and validation protocols) are provided in the Results section and detailed within the figure legends and tables. The value of n in all reported metrics denotes the number of test images in the respective dataset split. To ensure robustness, all experiments were run across three independent trials, with the mean and standard deviation (SD) reported for each metric. The center value represents the arithmetic mean, and dispersion is quantified using SD. Dataset partitioning was performed using fixed train/validation/test splits described in Section 4.1, and no manual exclusion of samples was carried out. Since only publicly available datasets were used, no randomization or stratification of biological subjects was applicable.

### Additional resources

No additional external resources, clinical registries, or supplementary websites are required beyond those listed in the “data and code availability” section.
