# Supplementary material for: Class-balanced dermoscopic lesion segmentation using MoG-LISA and optimized Swin-UNet via the GM-FDE framework
Source: iScience. 2026 Feb 13;29(3):115012. doi: 10.1016/j.isci.2026.115012 (PMC12969148; doi:10.1016/j.isci.2026.115012)
Supplement: Document S1. Methods S1 [file mmc1.pdf]

iScience, Volume *29*

Supplemental information

Class-balanced dermoscopic lesion segmentation using MoG-LISA and optimized Swin-UNet via the GM-FDE framework

S. Muthamil Selvan and R. Kavitha

Methods S1: Pseudocode description of the MoG-LISA augmentation and CB-SwinGMO optimization framework

ALGORITHM 1: GENERATION OF MOG-LISA FOR MINORITY-CLASS LESION SYNTHESIS

Input: *D, C_{min}, E, D, S, Augmentation threshold T_c.*

Output: D_{aug}: Class-balanced dermoscopic dataset.

1. ***Extract lesion-aware features*** *from each $x_i \in D$ using a deep encoder E, capturing asymmetry, border, color, texture, and size by* using Eqn. (13).
2. ***For*** *each class $c \in C_{min}$:*
   - **1.1 Identify** $Z_c = \{z_i \mid y_i = c\}$
   - **1.2 While** $|Z_c| < T_c$,
     - **do:**
       - Randomly sample feature vectors $z_a, z_b \in Z_c$
       - Perform covariance-preserving interpolation: *by* using Eqn.'s (15) to (17).
3. ***Decode*** *Z_{syn} using decoder D by* using Eqn. (18).
4. ***Apply lesion mask*** *$m = S(\hat{x})$ to enforce morphology-aware constraints.*
   post-process $\hat{x}$ by:
   - Convex hull enforcement on lesion boundary.
   - Edge refinement and border sharpness adjustment.
   - Texture-based variation using localized filters.
5. ***Conduct automated image quality validation:***
   - *a.* Blurriness, segmentation accuracy, structural integrity.
   - ***b.*** **If** fails,
     - discard $\hat{x}$.

     Else
     - include in D_{aug}.
6. Continue until each $c \in C_{min}$ meets the class-balanced threshold T_c.
7. Combine original and synthetic images:

$$D_{aug} = D \cup \{(\hat{x}_j, c)\}_{j=1}^{T_c - |Z_c|}$$

ALGORITHM 2: CB-SWINGMO OPTIMIZATION USING GM-FDEF PROCEDURE

Input: *$D_{aug}, P_t = \{\Theta_1^t, \Theta_2^t, \ldots, \Theta_N^t\}$, N, GMO and training parameters.*

Output: *P_{t+1}: Updated population with optimized Swin-UNet configurations.*

1. ***Initialization***
   Set generation $t = 0$; randomly initialize Swin-UNet configurations P_t.
2. ***Generate Intermediate Solutions:***
   Apply GMO operator to obtain Z_i^{t+1}, the intermediate configuration for each $i \in \{1, \ldots, N\}$, forming set Z_{t+1}.
3. ***Feedback-Driven Combination (GM-FDEF)***
   For each i:
   - Select historical best configuration $H_j^t \in P_t$.
   - Compute the new configuration:
     $$C_i^{t+1} = \alpha \cdot Z_i^{t+1} + \beta \cdot H_j^t$$
     where $\alpha = \frac{F\left(H_j^{(t)}\right)}{F\left(Z_i^{(t+1)}\right) + F\left(H_j^{(t)}\right)}, \beta = \frac{F\left(Z_i^{(t+1)}\right)}{F\left(Z_i^{(t+1)}\right) + F\left(H_j^{(t)}\right)}$
4. ***Form Candidate Pool***
   $Q_t = \{C_i^{t+1} \mid i = 1 \ldots N\}$
5. ***Merge Populations***
   $R_t = P_t \cup Q_t$
6. ***Pareto-Based Front Sorting:***
   Perform non-dominated sorting of R_t into fronts:
   $F_1, F_2, \ldots, F_l$
7. ***Survivor Selection:***

Initialize $S_t = \emptyset, i = 1$

Repeat:

$S_t = S_t \cup F_i$, $i = i + 1$

Until:

$|S_t| \geq N$

8. ***Population Update:***

   If $|St| = N$:
   - $P_{t+1} = S_t$

   Else:
   - Let F_l be the last overflow front
   - Select remaining $N - |St \setminus Fl|$ configurations from F_l using diversity potential *by* using Eqn. (30).
   - Combine to form P_{t+1}

9. ***Termination Check:***

   If stopping criterion is met (e.g., max generations or convergence):
   - Output P_{t+1}

   Otherwise:
   - Increment $t \leftarrow t + 1$, and repeat from Step 2.
